# Differential transcript isoform usage pre- and post-zygotic genome activation in zebrafish

**DOI:** 10.1186/1471-2164-14-331

**Published:** 2013-05-15

**Authors:** Håvard Aanes, Olga Østrup, Ingrid S Andersen, Lars F Moen, Sinnakaruppan Mathavan, Philippe Collas, Peter Alestrom

**Affiliations:** 1 BasAM, Norwegian School of Veterinary Science, 0033 Dep, Oslo, Norway; 2 Stem Cell Epigenetics Laboratory, Institute of Basic Medical Sciences, Faculty of Medicine, University of Oslo, Oslo, Norway; 3 Norwegian Center for Stem Cell Research, 0317Oslo, Norway; 4 Stem Cell and Developmental Biology, Genome Institute of Singapore, 60, Biopolis Street, #02-01, Genome 138672, Singapore

**Keywords:** Zebrafish, Mid-blastula Transition, Zygotic Genome Activation, Alternative Splicing, Transcriptional Start Site, 3’UTR

## Abstract

**Background:**

Zebrafish embryos are transcriptionally silent until activation of the zygotic genome during the 10^th^ cell cycle. Onset of transcription is followed by cellular and morphological changes involving cell speciation and gastrulation. Previous genome-wide surveys of transcriptional changes only assessed gene expression levels; however, recent studies have shown the necessity to map isoform-specific transcriptional changes. Here, we perform isoform discovery and quantification on transcriptome sequences from before and after zebrafish zygotic genome activation (ZGA).

**Results:**

We identify novel isoforms and isoform switches during ZGA for genes related to cell adhesion, pluripotency and DNA methylation. Isoform switching events include alternative splicing and changes in transcriptional start sites and in 3’ untranslated regions. New isoforms are identified even for well-characterized genes such as *pou5f1*, *sall4* and *dnmt1*. Genes involved in cell-cell interactions such as *f11r* and *magi1* display isoform switches with alterations of coding sequences. We also detect over 1000 transcripts that acquire a longer 3’ terminal exon when transcribed by the zygote compared to their maternal transcript counterparts. ChIP-sequencing data mapped onto skipped exon events reveal a correlation between histone H3K36 trimethylation peaks and skipped exons, suggesting epigenetic marks being part of alternative splicing regulation.

**Conclusions:**

The novel isoforms and isoform switches reported here include regulators of transcriptional, cellular and morphological changes taking place around ZGA. Our data display an array of isoform-related functional changes and represent a valuable resource complementary to existing early embryo transcriptomes.

## Background

During the first ten cell cycles after fertilization, the zebrafish embryo is transcriptionally silent and consists of undifferentiated and rapidly dividing blastomeres [1]. Initiation of transcription at the 10^th^ cell cycle is termed zygotic genome activation (ZGA). At the time of ZGA, development proceeds from a control by mRNAs synthesized during oogenesis and stored in the egg (maternal transcripts) to mRNAs produced by the embryo’s own genome. Following ZGA, blastomeres divide less frequently and more asynchronously, they start to differentiate and migrate to form the three germ layers of the gastrulating embryo [1-4]. Collectively, these transformations characterize the mid-blastula transition (MBT). These fundamental cellular and functional changes occurring during development make zebrafish an attractive model to study transcriptional changes governing between pre-MBT and post-MBT development.

Previous studies have shown essential roles of activation and degradation of maternal transcripts in regulating the MBT and ZGA [5-7]. Signaling pathways involving Bmp, Nodal, Fgf, Wnt and maternal β-catenin are essential for the formation of germ layers and body axis [8]. We and others have shown that the establishment of post-translationally modified histones on specific genomic sites and DNA methylation play a role in transcriptional regulation around the time of ZGA by patterning developmental gene expression [9-12]. However, although several early transcriptomes have recently been published [5,13,14], little is known on the isoform-specific dynamics governing developmental transitions around the MBT.

The notion that each gene can give rise to multiple mRNAs has evolved from being reported as a rare phenomenon [15,16] to include virtually all loci in man [17,18], and has been shown to be crucial for differentiation [19], development [20] and human disease [21]. Isoform switches, defined as a change in the isoform composition of gene products between two conditions (e.g. two developmental stages) are of particular interest since such events are critical for differentiation [19]. The differences between transcript isoforms can affect the coding sequence (CDS) and/or untranslated regions (UTRs) 3' or 5' of the CDS. The former is likely to affect protein function, while the latter may affect translational efficiency, mRNA degradation kinetics and spatial distribution of transcripts [22,23]. The mechanisms regulating splicing and determining the production of specific transcript isoforms have started to be unveiled and involve *cis*-elements and *trans*-acting factors, as well as epigenetic modifications in the proximity of spliced exons [24-26]. A genome-wide landscape of transcript isoforms synthesized during early development, and particularly of the switches occurring between maternal and zygotic isoforms at the time the embryo initiates its own transcriptional program, has been lacking and has hampered a comprehensive appreciation of the transcriptional dynamics occurring at the time of ZGA.

We have used an isoform prediction and quantification program, Cufflinks [27], to detect novel isoforms and quantify isoform-specific changes from RNA-sequencing (RNA-seq) reads before and after ZGA in zebrafish. We identify numerous novel isoforms related to shifts in transcription start site (TSS), alternative splicing (AS) events and transcription termination sites (TTS), when comparing transcripts of maternal and zygotic origin coming from the same gene. These include transcripts of genes involved in cell-cell interactions, pluripotency control and DNA methylation. Using H3K4 and H3K36 trimethylation (me3) data acquired by chromatin immunoprecipitation and high-throughput sequencing (ChIP-seq), we find that H3K4me3 can form relatively broad domains which cannot distinguish between closely spaced alternative TSSs, unless TSSs are linked to alternative promoters at distant locations. Skipped exons are enriched in H3K36me3 and H3K4me3, extending previous reports on the involvement of these histone modifications in the regulation of isoform usage.

## Results and discussion

### Mapping of RNA-seq reads achieved from combined use of two aligners

**Figure 1:**
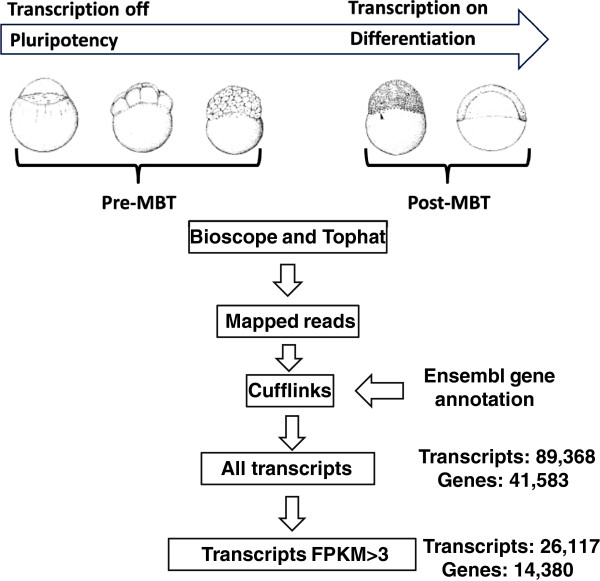
**Transcript isoform analysis pipeline.** Mapped reads from the egg and three pre-MBT samples (1-cell, 16-cell and 256-cell) were merged to represent the maternal transcriptome. This period is characterized by absence of transcription and pluripotent blastomeres. Two samples were merged to represent the first zygotic transcriptome, representing the embryo when cells start to differentiate and transcription has begun. The RNA-seq reads were mapped using two different aligners (Tophat and Bioscope) and Cufflinks was used to construct transcriptomes guided by Ensembl annotation. This resulted in a complete gene annotation set (‘all’) comprising both expressed and non-expressed genes and one dataset with robust >3 FPKM level of expression (FPKM > 3). Embryo illustrations are from Kimmel et al. (1995).

Using two different short RNA-seq read aligning programs, namely Bioscope (Life Technologies, USA, Carlsbad, CA) and TopHat [28], we remapped all reads generated by RNA-seq in a recently published study [5]. This ‘two aligner’ approach yields a higher number of mapped reads than each aligner alone and proves to be complementary compared to using either aligner separately (Figure S1 and S2 in Additional file 1). From our RNA-seq data, four datasets from pre-ZGA zebrafish developmental stages (pre-MBT) and two from post-ZGA stages (post-MBT) were merged, respectively generating one maternal transcriptome dataset, and one combined late maternal-early zygotic (henceforth termed zygotic) dataset (Figure 1). This strategy yields an opportunity to examine the embryonic transcriptome before and after ZGA, and provides more sequencing depth and thus higher accuracy in constructing transcripts. A total of 71.5 and 49 million RNA-seq reads were thus mapped from the pre-MBT and post-MBT developmental stages, respectively.

### Transcriptome assembly identifies novel genes and isoforms

**Figure 2:**
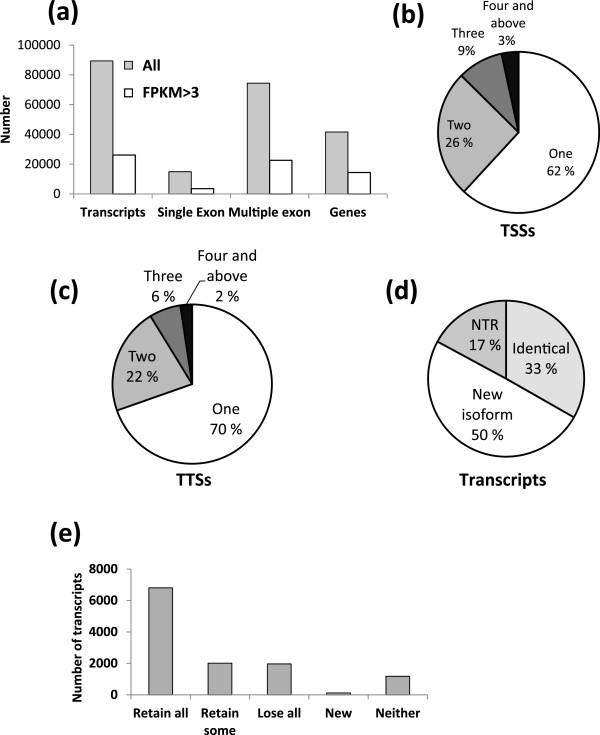
**Characterization of the pre-ZGA and post-ZGA embryo transcriptomes. (a)** Number of genes, transcripts and transcripts with one or more exons in the all and FPKM > 3 datasets. **(b)** Percentages of loci with 1, 2, 3 or more TSSs. **(c)** Percentages of loci with 1, 2, 3 or more TTSs. **(d)** Percentage of isoforms classified as NTR, identical (known) or new in the FPKM > 3 dataset. **(e)** Difference between the novel and annotated transcripts on functional domain(s) content. Most novel isoforms had retained all functional domains as compared to their closest matching annotated counterpart.

We used the Cufflinks software and associated tools [27,29] to assemble transcriptomes based on the mapped reads (Figure 1). Cufflinks was run in Reference Annotation Based Transcript assembly (RABT)-mode [30], using annotations from Ensembl as a guide (Zv9, version 64). We created two datasets. i) A set of all Ensembl annotations and all transcripts assembled was generated, referred to as the ‘all’ gene annotation; this set contains 89,368 transcripts from 41,583 genes (Additional file 2). ii) Expression values were obtained for the ‘all’ dataset (Additional file 3) and these were used to filter the ‘all’ gene annotation to generate a file of robustly expressed transcripts, defined as fragments per kilobase per million mapped fragments with a value >3 (FPKM > 3) (Additional file 4). This dataset contains 26,117 isoforms from 14,380 genes (Figure 1). Of these transcripts, the vast majority (22,600) have multiple exons (Figure 2a). These multiple-exon transcripts arise from 10,863 genes (Figure 2a), thus multi-exon loci produce on average nearly 2 isoforms. Altogether, we identify 22,453 TSSs (Figure 2b) and 20,562 transcription termination sites (TTSs) in the FPKM > 3 gene annotation dataset (Figure 2c). This indicates that a large fraction of the isoforms arise from differential use of TSSs or TTSs. However, the fact that isoforms from the same locus with different TSSs also frequently differ in their exon content is a reflection of Cufflinks’ parsimonious nature, because Cufflinks proposes as few transcript models as necessary to explain the aligned reads [27].

To identify novel isoforms and genes, the predicted isoforms in the FPKM > 3 dataset were compared to Ensembl annotations (Zv9, version 64). This classifies 8,108 of the transcripts as identical, 12,100 as new isoforms, and 4,174 as novel transcribed regions (NTRs) i.e. possibly novel genes (Figure 2d). This analysis indicates that the assembled zebrafish transcriptome contains numerous novel transcripts, some of which may be encoded by novel genes.

### Functional differences between annotated and novel transcripts

The large number of novel isoforms warranted further validation and characterization. We investigated the coding potential of new isoforms and found that nearly all have an open reading frame (ORF) (11,627/12,100; 96%) of >50 amino acids in length (starting with an AUG start codon and ending with a stop codon). Increasing the ORF length requirement results in a slight decrease in the number of ORFs detected (100 amino acids, 89%; 150 amino acids, 81%).

We then compared both the transcript sequence and the inferred coding sequence between novel isoforms and their closest matching annotated transcript from the same locus (as defined by Cufflinks). On average, the novel isoforms have shorter coding sequences than the corresponding annotated coding sequences with a mean difference of −230 bp (median −109 bp). Of 11,529 comparisons, 1,695 of the novel isoforms differ by one amino acid or less, indicating identical coding sequences (this corresponds to 15% of the transcripts with an ORF of ≥50 amino acids). Transcript isoforms with no difference in coding sequences length have on average longer 5’ and/or 3’ UTRs, with a mean length difference of 549 bp (median 256 bp). Therefore, the novel isoforms can be broadly divided into two groups, those with no change in coding sequence length but with longer 5’ and/or 3’ UTR, and those with an alteration in coding sequence length.

We next examined any functional effect these transcript isoforms may have, and used the predicted protein sequences for a domain scan using Pfam, a database of protein families [31]. The detection of a protein domain has been shown to be suggestive of protein functionality [32]. Of the novel isoforms analyzed, 8,949 (74%) contain a sequence with high similarity to an identified protein domain. In comparison, the closest matching annotated transcripts (n = 5,908) have a substantially larger fraction of Pfam domain-containing sequences (5,196; 88%). Further, most of the novel isoforms encode the same functional domains as the annotated isoforms (Figure 2e). However, a substantial fraction loses either all functional domains (n = 1971), or some of them (n = 2017; Figure 2e). Also, a small subgroup (n = 123) of the novel isoforms contains functional domains absent from the annotated isoform (Figure 2e). Taken together, these results suggest that the majority of the novel transcript isoforms are functional, with roles related to their annotated counterparts. However, some lose or gain functional domains, suggesting that they might exhibit different biological functions.

### TSS usage and switching

**Figure 3:**
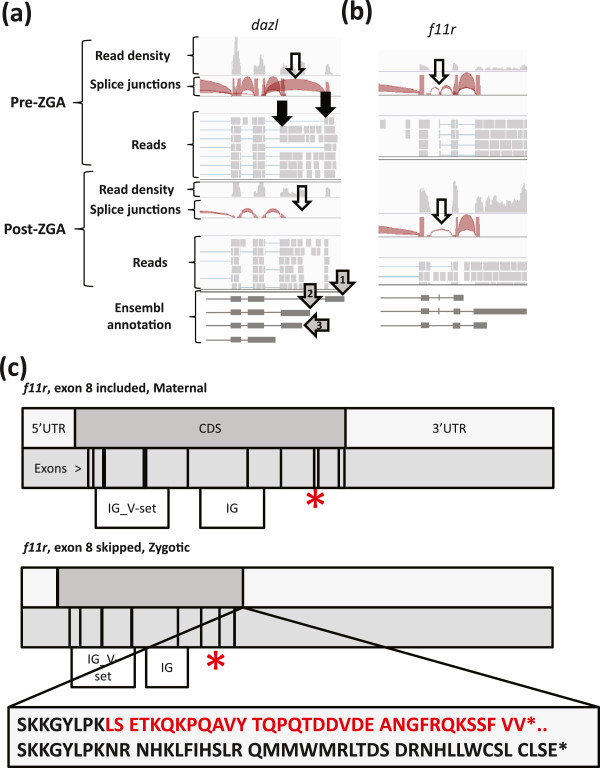
***Dazl *TSS switching and *f11r *exon skipping switch. (a)** A screenshot from Integrative Genomics Viewer shows that *dazl* has been transcribed from at least two TSS’s in the maternal transcriptome; grey arrows 1–3 at bottom indicate Ensembl annotated TSSs. Black arrows in the upper panel indicate reads supporting promoter 1 (rightmost black arrow) and promoter 2/3 (leftmost black arrow; our data cannot distinguish between TSS 2 and 3). A white arrow in the upper panel points at a splice junction ‘bridge’ (red), symbolizing the splice junction for the most distal first exon. The thickness of splice junction symbols is correlated with the number of reads supporting the junction. Post-ZGA transcripts lack the most 5’ exon as can be observed from the absence of reads supporting the splice junction originating from this exon (white arrow, bottom panel). **(b)** Pre-ZGA an *f11r* isoform with exon 8 included is present (white arrow, top panel). Post-ZGA a different *f11r* isoform, this one with exon 8 lacking, has been transcribed (white arrow, bottom panel). **(c)** The *f11r* exon skipping (red asterix) leads to a frame shift resulting in a 6 nt longer ORF, including the removal of two C-terminal valine residues. Two functional domains, immunoglobulin V-set domain (PF07686) and immunoglobulin domain (PF00047) (white bars) are intact in both splice isoforms.

Transcription is initiated at TSSs and differential usage of TSSs is often associated with tissue-specific expression or distinct protein products [33]. Using Cufflinks, we identify 513 loci (q-value < 0.001; Additional file 5) with a change in TSS usage between pre- and post-ZGA stages. A typical example is the isoforms of *dazl* encoding an RNA-binding protein crucial for germ cell development, oocyte-to-zygote transition and maintenance of pluripotency [34-36]. The biological function of the Dazl protein is at the level of regulation of translation [37] and recent experiments indicate that Dazl protects transcripts from degradation, possibly through antagonizing the de-adenylating effect of miRNAs [38]. Our data show that the transcript isoforms of *dazl* are expressed from at least two promoters maternally (Figure 3a; promoter 1 = grey arrow 1 (most upstream); promoter 2 = grey arrows 2 and 3, black arrows in the top panel point towards reads supporting expression from both TSSs pre-ZGA). In the post-ZGA transcriptome, the lack of reads supporting usage of promoter 1 (Figure 3a; white arrow, bottom panel) demonstrates degradation of the maternal-specific 5’ distal isoform during the MBT (Figure 3a). RT-qPCR confirms the trend observed by RNA-seq (Figure S3 in Additional file 1). Both *dazl* isoforms are present in Ensembl (pre-ZGA: ENSDART00000147911; post-ZGA: ENSDART00000137590). The CDS of the maternal-specific transcript displays a 17 amino acid shorter N-terminus compared to the zygotic transcript. However, the functional implication of this difference is not straightforward because the RNA-recognition motif of the Dazl protein is located 30 amino acids downstream and is not affected, unless induced by configuration change. Considering the diverse and temporarily restricted roles of Dazl in germ cell development and oocyte-to-zygote transition [34,35], it is tempting to speculate that the maternal and zygotic isoforms may be responsible for two biological processes: the ‘maternal’ version may act in the oocyte-to-zygote transition, whereas the zygotic variant may act in germ cell development.

Among the 513 genes which display a change in TSS pattern between the pre- and post-ZGA, *dazl* represents a subset where the TSSs are defined by different first exons. This type of TSS switch is likely to affect developmental functions as illustrated with *dazl*s changes in the 5’UTR and N-terminus of the protein.

### Epigenetic regulation of TSS usage and expression

H3K4me3 enrichment over the TSS is considered as a histone modification associated with transcription permissiveness. While virtually all expressed genes are marked by H3K4me3 at the TSS [39,40], many genes that are not expressed also harbor H3K4me3 [9,41]. H3K27me3 promoter occupancy is on the other hand associated with transcriptional repression [42]. To identify a relationship between TSS usage and potentially associated chromatin marks, we used published ChIP-seq peak calling data for H3K4me3 and H3K27me3 in post-MBT stage embryos [13], and investigated the correlation between these histone modifications and the TSSs mapped in our study.

We find that a subset of 22,534 TSSs from 13,542 genes from our ‘all’ gene annotation dataset are enriched in H3K4me3. Most peaks (18,547/23,778; 78%) were within ±500 bp of a gene. The number of TSSs in the dataset and the number of peaks covering them indicates that multiple TSSs are included within each H3K4me3 peak, as exemplified by *dazl* and *sall4* (Figure S4a,b in Additional file 1). On rare occasions, we observe that H3K4me3 peaks are located over the most frequently used TSS (major TSS) (e.g. *actl6a*; Figure S4c in Additional file 1). Therefore, at present H3K4me3 peaks cannot be used to predict the major TSS since the mark can overlap multiple TSSs. More accurate determination of TSS usage, e.g. using approaches such as cap analysis of gene expression (CAGE) together with H3K4me3 enrichment data may improve the usefulness of this histone modification in predicting major active start sites among closely located TSSs.

Our RNA-seq data show that 21% of the H3K4me3-marked genes are expressed at very low levels, with 2,822 loci displaying an FPKM value below 1 in both pre- and post-ZGA samples (Additional file 3). For the subgroup of bidirectional promoters identified using the H3K4me3 peaks (n = 1,433 promoter pairs) (see Methods) we find no significant correlation in expression between the bidirectional gene pairs (Pearson’s product–moment correlation <0.01), again supporting H3K4me3 as a mark for predisposition of promoter activation rather than having a direct activating effect, even when presumably sharing the same promoter. However, on average, H3K4me3-marked genes are more strongly expressed than genes without H3K4me3, as described earlier for RefSeq genes (data not shown) [10].

An important question is whether epigenetic marks can regulate particular subgroups of genes, functionally and spatially. To address the first question, analysis in DAVID of gene ontology (GO) terms for biological functions were obtained using H3K4me3- and H3K27me3-marked genes. This was significant only for one term for the H3K4me3-marked genes; cell cycle (FDR < 0.001; Additional file 6). This is in contrast to H3K27me3-marked genes which were enriched for 21 GO terms with the same cut off (i.e. regulation of transcription, embryonic morphogenesis, pattern specification process and regionalization; Additional file 7). Thus, whereas H3K4me3 mark TSSs of genes linked to many biological functions, H3K27me3 marks mostly the TSSs of genes involved in developmental functions. These results are consistent with a H3K4me3 pre-patterning of promoters of a specific subset of genes pre-MBT (involved in housekeeping and homeostatic functions), and with a view of H3K27me3 repressing developmental genes prior to their activation at or after the MBT [9,10].

Another intriguing question is whether TSS marking with both H3K4me3 and H3K27me3 exists (so-called ‘bivalency’). We utilized the extensive annotation database of where zebrafish genes are expressed anatomically (anatomical ontology provided by the zebrafish model organism database; ZFIN [43]) to bring further clarity to this issue. H3K4me3-marked genes are mostly expressed in the entire organism, in contrast to H3K27me3-marked genes which are predominantly annotated with expression in distinct tissue types (in particular central nervous system structures; Additional file 8 and Additional file 9). This is consistent with H3K27me3 analysis of dissected *Xenopus* embryos [44], and not supportive of bivalency in zebrafish embryos, as others have suggested [9].

Taken together, our results show that during zebrafish development, H3K4me3 is a mark of TSSs independent of transcription status. The marked genes show no pronounced tissue-specific preferences and few specialized biological function as a group. In contrast, H3K27me3 marked genes have specific biological functions and are expressed in specialized cells. Our results are in line with the view that H3K4me3 contributes to a transcriptionally permissive chromatin environment for TSS usage; however, the repressive marks seem to contribute to lineage commitment and tissue-specific differentiation [45]. The use of ChIP-seq data and a comprehensive gene annotation based on existing and novel annotations confirm the trends from previous studies based on older annotation and ChIP-chip data [9,10].

### Shifts in alternative splicing during the switch from maternal to zygotic transcripts

Alternative splicing (AS) allows the production of multiple mature mRNAs from the same primary transcript, increasing genetic diversity of a genome by several-fold. We used Cufflinks to identify shifts in AS isoform patterns before and after the ZGA. A total of 2,160 primary transcripts exhibited a significant change in the splice isoform composition (examples include *foxh1*, *hdac5*, *dnmt3* and *gsk3a*; Additional file 10). Quality control by visual inspection revealed mostly subtle changes among the significantly changing primary transcripts. Importantly we find, also by visual inspection, that several obvious isoform switches do not pass the significance criteria of Cufflinks. For example, we report *f11r*, encoding a trans-membrane adhesion protein found in association with tight junctions [46,47], which displays a switch between the maternal and zygotic stages, with exon 8 skipped only in the zygotic transcripts (Figure 3b). Using reverse transcription (RT)-qPCR, we validated the expression pattern of *f11r* (Figure S5 in Additional file 1). Exon 8 of *f11r* is only 13 bp long and is included in the maternal isoform of the *f11r* transcript (Figure 3c, upper panel). Skipping of exon 8, detected in the zygotic transcriptome (Figure 3c, lower panel), causes a frame-shift with an alteration of the C-terminal 36 amino acids; however two known functional domains in the F11r protein are not directly affected, although indirect structural effects cannot be excluded (Figure 3c). The change in the intracellular C-terminal portion of the protein includes the removal of two terminal valine residues which are involved in binding to PDZ-domain containing proteins [46]. In zebrafish, interference with F11r function causes defects in migration at later stages of development [48]. Interestingly, the shift in *f11r* isoform usage between pre- and post-MBT stages coincides with the initiation of cell migration taking place after the MBT [1,2]. It is tempting to speculate that attenuation of binding potential to PDZ domain-containing proteins enables cells to alter their migration patterns [49,50]. However, functional studies are required to test this hypothesis.

From our dataset, it appears therefore that thousands of splice isoform switches occur between the maternal and zygotic transcriptomes; nonetheless, it is important to emphasize that the majority constitutes subtle changes rather than obvious functional on/off events.

### Quantification of AS events

**Figure 4:**
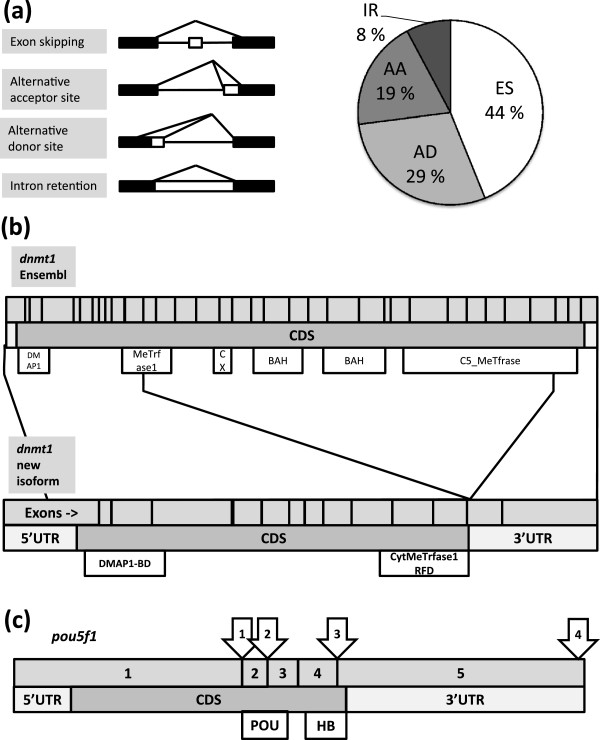
**Alternative splicing in the early embryo. (a)** The frequency of 4 different alternative splicing events: Exon skipping (ES), alternative acceptor site (AA), alternative donor site (AD) and intron retention (IR). ES is the most frequent AS event. In most ES events multiple exons are skipped. **(b)** Schematic representation of an annotated version (top) and a new (bottom) isoform for *dnmt1*. Black lines between the isoforms represent the conserved area. Functional domains (white bars) were defined by Pfam: DMAP1 binding domain (DMAP1-BD, PF06464), Cytosine specific methyltransferase replication foci domain (Cyt MeTrfase1 RFD, PF12047), CXXC zinc finger domain (“CX”: Znf CXXC, PF02008), Bromo-adjacent homology domain (BAH, PF01426), C-5 cytosine-specific DNA methylase domain (C5_ MeTfrase, PF00145). In the new isoform 19 exons are skipped, and all functional domains except a DMAP1 binding domain are lost. The novel isoform also have a longer 5’UTR relative to the annotated version. **(c)** Novel *pou5f1* isoforms. The annotated version of *pou5f1* has two Pfam domains; Homeobox domain (HB, PF00046) and POU (PF00157). We identify 3 novel alternative acceptor sites (arrows 1–3) all within the CDS of *pou5f1*. The first leads to a 3 nt deletion and removal of one glutamic acid, upstream of the POU; the second a 19 nt insertion causing a frame shift and a truncated Pou5f1 protein with both functional domains lost; the third event gives a 4 nt deletion which truncates the HB. We also detect a longer 3’ UTR post-ZGA (arrow 4).

We used the software ASTALAVISTA [51] to quantify the frequency of four different types of AS events; exon skipping (ES), alternative donor (AD) and acceptor (AA) sites and intron retention (IR) (Figure 4a). Using the FPKM > 3 transcript subset, we identify a total of 10,950 AS events linked to 3,843 loci (36% of the multi-exon loci). AA and AD sites make up 2,107 (19%) and 3,185 (29%) of these events, respectively, and we find 852 IR and 4,806 ES occurrences (Figure 4a). Noteworthy, only 39% of the ES events involve the skipping of a single exon, while the majority of these events involve the skipping of ≥2 exons (61%).

An example of the latter includes *dnmt1*, encoding DNA methyltransferase 1 responsible for maintenance of DNA methylation patterns, particularly during development (reviewed in [52]). *dnmt1* has approximately 34 exons with three isoforms annotated in zebrafish; however, our analysis detects several additional isoforms (Figure S6 in Additional file 1). Cufflinks predicts 9 *dnmt1* isoforms (FPKM > 3), but without full-length sequence data the number of biologically relevant isoforms remains uncertain. One new isoform of *dnmt1* is robustly expressed maternally (Additional file 3) and truncated due to skipping of 19 exons (validated by cDNA cloning and sequencing; data shown in Additional file 11). This skipping event removes most of the functional domains involved in DNA methyltransferase activity, but it retains a DMAP1 binding domain (Figure 4b). This domain binds the co-repressor DMAP1 and thereby confers DNMT1 with transcriptional repressor activity [53,54]. Interestingly, in *Xenopus* it has been shown that Dnmt1 is needed to repress transcription prior to the MBT [55] and this function does not rely on the cytosine methyltransferase catalytic activity of Dnmt1 [56]. Zebrafish may therefore be a valuable candidate to shed more light on the functional role of *dnmt1* and its isoforms. The novel *dnmt1* isoforms discovered in this study may bring clarity to the enigmatic issue of transcriptional repression during cleavage stages in zebrafish [11,57] and other species [58].

For the pluripotency-associated gene *pou5f1* (also known as *oct4*) we identify several new splicing events, all of which were confirmed by cDNA cloning and sequencing (sequences are shown in Additional file 12). In zebrafish, there is only one annotated *pou5f1* transcript, while in human three arise from AS [59]. We observe novel *pou5f1* AS events originating from alternative acceptor sites at exons 2, 3 and 5 (Figure 4c; Figure S7 in Additional file 1). The first event leads to deletion of a nucleotide triplet coding for glutamic acid (validated by one cDNA clone sequence out of 12 random clones analyzed), yet with no effect on the functional domains. The removal of a charged amino acid is however likely to affect the three-dimensional structure of Pou5f1, although functional consequences remain unknown at this stage. The second AS event is located 19 nt prior to the annotated exon 3 (validated by one cDNA clone sequence). This leads to a frame-shift and a truncated Pou5f1 protein, removing both functional domains. Lastly, a 4 nt deletion causes a frame-shift in the exon 5 (validated by 5 of 12 cDNA clones sequences, suggesting higher abundance of this isoform) and leads to a truncation of the homeobox domain. Of note, we also find novel isoforms for other genes important for pluripotency, such as *klf4*, *sall4* and *nanog*. This indicates that several well-studied genes with central functions in pluripotency are still not fully annotated in zebrafish, and highlights a likelihood of uncovering novel intricacies about pluripotency control mechanisms.

### Epigenetic marks associated with exon skipping

AS predominantly occurs co-transcriptionally, which may influence the regulation of splicing by changes of epigenetic signatures such as histone modifications and DNA methylation [24,25,60]. We used ChIP-seq to map H3K36me3 occupancy in post-ZGA embryos, and used published peak calling data for H3K4me3 and H3K27me3 [13] to investigate any association between these modified histones and single ES events. We extracted the number of reads spanning each skipped exon (this is direct evidence for skipping of an exon; see Methods); 1,668 single ES events have one or more reads supporting the skipping event, of which most only with a few reads spanning them. Focusing on 446 exons with >5 reads supporting the skipping of these exons at the post-MBT stage, we examined peaks of H3K4, H3K27 and H3K36 trimethylation in their vicinity (see Methods). H3K36me3 peaks and skipped exons co-localize in 194 of the 446 exons (some exons overlap with multiple H3K36me3 peaks), which represent a slight overrepresentation relative to a random set of regions (~145 overlaps on average when 446 exons were drawn at random from all exons a thousand times; Chi-squared test, p < 0.001). H3K4me3 is also slightly overrepresented (113 overlaps versus 71 at random, p < 0.001), whereas H3K27me3 is not overrepresented over skipped exons (~35 peaks overlapped both skipped and non-skipped exons).

**Figure 5:**
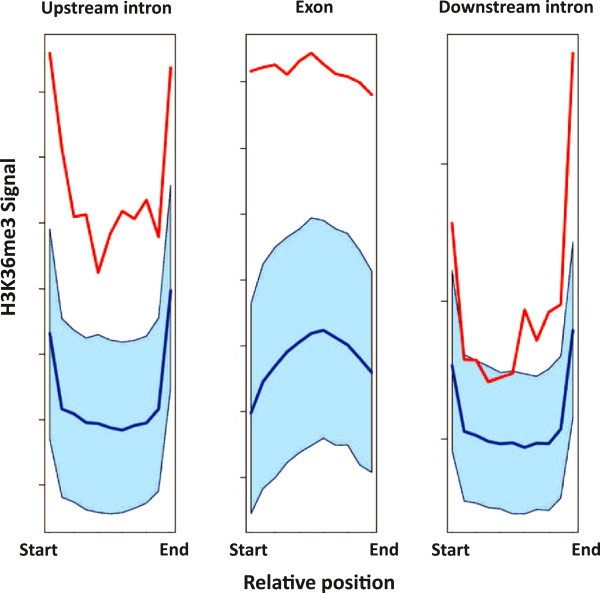
**Metagene projections of average H3K36me3 enrichment levels over skipped exons and surrounding introns.** Metagenes based on H3K36me3 levels for upstream introns, skipped exons and downstream introns show increased H3K36me3 levels in the upstream intron and the exon, but only to minor extent in the downstream intron, as compared to background. Red line = transcripts with skipping event. Blue line = random set of introns and exons with confidence interval (light blue shaded region). The X-axis is in percentage of total length (start = 0% and end = 100%) and the Y-axis is the H3K36me3 signal normalized against input control and the total number of reads in the sample.

From metagene sections of average enrichment profiles containing the skipped exon as well as introns upstream and downstream of the skipping event, we find that both the upstream intron and the skipped exon display higher levels of H3K36me3 than average, determined from random selections of the entire set of introns and exons (Figure 5). Introns localized downstream of the skipped exons only show slightly higher H3K36me3 enrichment values than the average. Since H3K36me3 associates with expressed genes [61], a possible explanation for H3K36me3 enrichment on the skipped exons may be that the exons are only skipped in a subpopulation of cells in the embryo, reflecting distinct transcript profiles in individual cells. Isoforms with the exon included can thus be more prevalent and account for the observed enrichment in H3K36me3. However as expected, globally in the cell populations examined, the skipped exons have on average substantially lower number of reads mapped to them than the rest of the exons (Figure S8 in Additional file 1). This shows that the H3K36me3 enrichment at skipped exons is not due to expression of an isoform with the exon included in the majority of the cells. Collectively, our observations are consistent with a role of H3K36me3 and H3K4me3 as possible facilitators, rather than determinants, of increased exon skipping [24,62].

### Longer 3’UTRs in zygotic than in maternal transcripts

**Figure 6:**
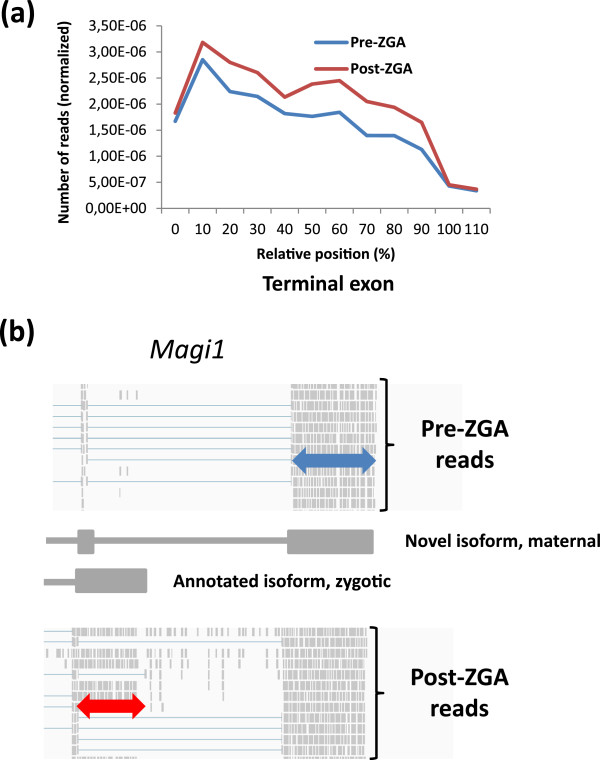
**Extended 3’ terminal exons and alternative last exons. (a)** Metagene representation of RNA-seq reads for the last exon in a set of transcripts detected with more coverage in the proximal half of the terminal exon post-ZGA (red line) as compared to pre-ZGA (blue line). X-axis is in percentage of total length and Y-axis is number of reads normalized against total number of reads in the samples. **(b)** The gene *magi1* displays a change in the expression of its isoforms pre- and post-ZGA with an alternative 3’UTR appearing post-ZGA (red double-arrow). Prior to the ZGA a novel isoform is expressed (blue double-arrow). This isoform is expressed also after the MBT but then together with the novel isoform.

The TTS can be regulated either through termination of transcription [63] or via the 3’-end processing machinery [64]. Regardless of the mechanism, sequence motifs in the 3’UTR play an important role in mRNA stability, spatial distribution and translational efficiency [65]. We observe that zygotic transcripts frequently have longer 3’UTRs than corresponding maternal transcripts, as exemplified by *pou5f1* (Figure 4d, arrow 4; black and white arrow in Figure S7 in Additional file 1). To investigate the genome-wide frequency of longer 3’UTR in post-ZGA relative to pre-ZGA embryos, we extracted all last exons and counted the number of reads mapping to the first and second half of the last exon. Using these counts, we calculated a numeric score where higher values indicate a shift towards a longer 3’ terminal exon post-ZGA (see Methods). We find that 1,249 last exons increase in length post-ZGA relative to pre-ZGA (Additional file 13). A metagene projection for these 1,249 last exons using RNA-seq coverage confirms the detection of more reads in the last half of the exons post-ZGA (Figure 6a). The observation of longer 3’UTRs can be interpreted in light of a recent report of shorter 3’UTRs of highly expressed transcripts [66]. We may infer from this report and our results that post-ZGA embryos produce transcripts with a more diversified control of turn-over kinetics, together with spatial and cell-type specific expression, than the oocyte. In *Drosophila melanogaster*, extension of 3’UTRs after the maternal-to-zygotic transition (coinciding with the MBT) have been reported [67]. Similarly in the mouse, the 3’UTR becomes progressively longer during embryonic development [68]. Furthermore, while this manuscript was in preparation, Ulitsky and colleagues [69] reported extended 3’UTRs in 419 genes after ZGA in zebrafish embryos, using poly(A)-position profiling by sequencing. Thus our findings validate previous reports in zebrafish and other species.

In addition to the extension of last exons, we also observe alternative last exon usage, which may lead to changes in the CDS, the 3’UTR or both. An example of the latter is apparent in *magi1*, encoding a PDZ domain protein; due to a switch in last exon usage between pre- and post-ZGA, both the CDS and the 3’UTR are changed (Figure 6b; see also RT-qPCR validation in Figure S9 in Additional file 1). The 3’UTR of the post-ZGA-specific isoform contains three additional sequence domains absent in the maternal version, namely an internal ribosome entry site (IRES), a sex-lethal binding site (SXL-BS) and a K-box element. The latter two are known to often co-occur to create spatial and temporal expression patterns through negative regulation of translation [70,71]. In addition, although the PDZ domains are not directly affected, alteration in protein sequence might affect the protein 3D-structure and in particular the fifth PDZ domain located proximal to the C-terminus [72]. Interestingly, Magi1 interacts with β-catenin [73], another regulator of cell adhesion crucial for left-right asymmetry of the embryo [8,74]. Each PDZ domain often displays distinct protein specificity and strikingly, β-catenin binding to MAGI1 depends on the 5^th^ PDZ domain, thus potentially linking the isoform switch reported in our study to the regulation of Wnt signaling through β-catenin [75].

## Conclusions

The transcriptomes assembled in this study contain a high degree of complexity pertaining to TSSs, AS and TTSs. Most of the described novel isoforms encode ORFs with predicted significant functional protein domains, suggesting functional and valid transcripts with a role in development. We report several loci which display a switch-like behavior in isoforms expressed pre-ZGA and post-ZGA stages; these notably include *dazl* (TSS switch), *f11r* (exon skipping) and *magi1* (alternative last exon usage). Their changes in expression pattern coincide with key developmental processes such as onset of transcription upon ZGA, cell speciation and migration, suggesting functional roles for these isoforms. We also identify validated novel isoforms for *dnmt1* and *pou5f1*, important genes in development and pluripotency control. Moreover, we find increased length of the 3’ terminal exon and 3’UTR after the ZGA in over 1,000 transcripts. Analysis of histone marks suggests that H3K4me3 has little value in marking the major TSS in loci with several start sites. However, we find higher levels of H3K36me3 over skipped exons relative to all exons, suggesting a role of H3K36me3 in zebrafish AS control.

## Methods

### Bioinformatics

Bioinformatic analyses relied heavily on custom python scripts and the use of publically available tools such as SAMtools [76] and BEDtools [77], as well as general data handling in R [78]. Python scripts critical for the analyses are available as supplementary material (Additional file 14). Data were visualized in the Integrative Genomics viewer [79].

### RNA-seq mapping and transcriptome assembly

Embryo collection and RNA sequencing was performed as previously described [5]. Raw data files are available from NCBI’s short read archive (GSE22830). Reads were mapped using Bioscope 1.3 (with default settings) and TopHat 1.3.1 (with default options except: -a = 5, -I = 50, -F = 0). The resulting SAM-files were merged and a python script (best_alignment.py) used to ensure that a read were only present once. We performed transcriptome assembly using Cufflinks (version 1.2.1), with Ensembl annotation as guide (Zv9, annotation version 64), using default settings except; F = 0.0, -j = 0.05, -A = 0.08, --3-overhang-tolerance = 100. Additional programs in the Cufflinks pipeline were used to merge files, compare transcripts to known annotation and detect differences between the stages [80]. We created additional datasets using scripts; FPKM > 3 using extract_gtf.py, TSS’s using get_tss_bed.py and TTS using get_tts.py.

### Sequence analysis

We extracted transcript sequences using gffread in Cufflinks. These were subjected to ORF prediction using getORF (with settings: -minsize 150, -sreverse_sequence No, -find 1) from the EMBOSS suite [81]. The resulting protein sequences were processed by scripts and R handling to retrieve the longest predicted ORF. We used Pfam to identify functional domains (Pfam A) [31] and domains considered significant by Pfam were used. A script (‘domain_compare.py’) was used to identify if domains were retained, lost or gained relative to the closest matching transcript. Detection of motifs in the 3’UTR was performed using UTRScan [82].

### Alternative splicing

We used ASTALAVISTA version 2.2 [51] to quantify alternative splicing events. This program takes as input a GTF file (FPKM > 3) and outputs all splicing events in the annotation together with a code representing type of event (exon skipping, intron retention, alternative acceptor site, alternative donor site). We used python scripts to extract and count events from the ASTALAVISTA output (summary.py and events.py).

### Detection of length changes in last exon

We designed a database of non-redundant last exons (get_last_exon.py) and after initial filtering (exons > 400 bp, FPKM > 3 in both samples, n = 10,349 last exons) we used a script (find_extended_utrs.py) to idenitfy exons with a length change between pre- and post-ZGA. This was achieved by counting the number of reads in the first and latter part of the exon. A score was calculated as follows:

$$Q=\left(p2–p1\right)*\log\left({\mathrm{FPKM}}_{\mathrm{pre}}+{\mathrm{FPKM}}_{\mathrm{post})}/2\right)$$

where p1 and p2 are the proportions of reads in the last part of the exon relative to the first part pre- and post-ZGA, respectively. Exons with a shift towards more reads in the last part post-ZGA will have a positive value. We weighted this value using the log-transformed average of the exons FPKM values pre- and post-ZGA, assuming that higher coverage gives a more robust estimate of change.

### Analysis of ChIP-seq data

Embryos for ChIP-seq were collected at 5.3 hpf and chromatin prepared as described [83]. In accordance with institutional, national and international guidelines for early stage (0–5.3 hpf) zebrafish embryos no ethics committee approval was needed for any of the experiments performed in this study. Immunoprecipitation was performed using H3K36me3 (Diagenode 058–050, Denville, NJ) and ChIP enriched DNA and input samples prepared for sequencing according to Illumina standard protocol (#11257047 Rev.A). ChIP-seq reads were mapped using BWA [84] and peaks detected using CCAT 3.0 (Settings: fragment size = 200, sliding window size = 500, minimum score = 3.0) [85]. Intron and exons from single exon skipping events identified by ASTALAVISTA were extracted with a script (extract_skipped_exon.py) and BEDtools used to count the number of reads supporting each event. Events with >5 reads post-ZGA were considered further. We created databases of all introns and exons using the ‘all’ annotation. The number of overlapping peaks and skipped exons (>1 bp overlap between peak and exon skipping event: from start upstream intron to stop downstream intron) were calculated using BEDtools and the random control using a script (bootstr_rand.py). We utilized Repitools to construct metagens [86]. H3K4me3 and H3K27me3 peak data were from a recently published paper (Pauli et al., 2012). We identified potential bidirectional promoters using ChIPpeakAnno [87] with the H3K4me3 data [13] and the ‘all’ gene annotation file. The TSS of either gene in the pair had to be within 1000 bp of the middle of the H3K4me3 peak.

### PCR and cloning

Newly assembled and annotated isoforms were validated by RT-(q)PCR. RNA from pools of embryos at different stages was isolated using TRIzol reagent followed by RNA cleanup using Qiagen RNeasy MiniKit (Hilden, Germany) as previously described [88]. Control RNA of kanamycin (Promega #C1381, Madison, Wisconsin) was added prior to the RNA extraction and used as the reference control by qPCR. RNA was reverse transcribed using iScript Select cDNA synthesis Kit (BioRad #170-8896, Hercules, CA) according to the manufactures instructions. Primers were designed to cover specific sequences resulting from alternative splicing (*f11r*), alternative TSS (*dazl*), and alternative last exon usage (*magi1*) (for details see Supplementary Table 1 in Additional file 15). The dynamics of the isoforms was confirmed by RT-qPCR. qPCR was performed with the iCycler MyiQ real time PCR detection system and SYBR Green (BioRad, Hercules, CA). Primers pairs gave no signal in PCRs lacking template (data not shown). Relative expression was determined by the ∆∆-C_T_ method. For *pou5f1*, both primers were designed in the sequence common for the predicted isoforms (Supplementary Table 1 in Additional file 15). Presence of the isoforms at 3.5 hpf was confirmed by TOPO TA cloning (Invitrogen K45-0001, Carlsberg, CA) and subsequent sequencing of randomly selected clones.

### Data access

ChIP-seq data for H3K36me3 and input are available under [NCBI GEO GSE40629]. Our RNA-seq data are available under [NCBI GEO GSE22830].

## Abbreviations

RNA-seq: RNA-sequencing; ChIP-seq: Chromatin immunoprecipitation sequencing; ZGA: Zygotic genome activation; MBT: Mid-blastula transition; CDS: Coding sequence; UTR: Untranslated region; TSS: Transcription start site; AS: Alternative splicing; TTS: Transcription termination site; FPKM: Fragments per kilobase per million mapped fragments; ORF: Open reading frame; GO: Gene ontology; FDR: False discovery rate; ES: Exon skipping; AD: Alternative donor site; AA: Alternative acceptor site; IR: Intron retention.

## Competing interests

The authors declare that they have no competing interests.

## Authors’ contributions

HA designed the study, wrote the manuscript, made the figures and performed all bioinformatics analysis, including writing python scripts. OØ performed RT-qPCR, cDNA cloning and sequencing, contributed to figures and the manuscript. ISA performed ChIP experiments. LFM collected embryos, isolated RNA and made figures. SM designed the study, initiated the RNA-seq experiment and generated all the basic data. PC supervised ChIP-seq work, designed the study and wrote parts of the manuscript. PA designed the study, wrote the manuscript and supervised the work. All authors approved the final manuscript.

## Supplementary Material

Additional file 1 Supplementary figures 1–9. Click here for file

Additional file 2 Non-redundant gene annotations file containing a merger of all Ensembl and assembled transcripts. Click here for file

Additional file 3 Expression values pre-ZGA and post-ZGA for all isoforms in the ‘all’ gene annotation. Click here for file

Additional file 4 Gene annotation for robustly expressed isoform (>3 FPKM pre-ZGA or post-ZGA). Click here for file

Additional file 5 Test results for whether loci with multiple TSSs exhibited a shift in TSS usage between pre-ZGA and post-ZGA. Click here for file

Additional file 6 GO term analysis results from DAVID of genes marked with H3K4me3. Click here for file

Additional file 7 GO term analysis results from DAVID of genes marked with H3K27me3. Click here for file

Additional file 8 Anatomical ontology results from DAVID of genes marked with H3K4me3. Click here for file

Additional file 9 Anatomical ontology results from DAVID of genes marked with H3K27me3. Click here for file

Additional file 10 Results from a statistical test of whether genes exhibit a significant change in alternative splicing between pre-ZGA and post-ZGA. Click here for file

Additional file 11 **Results from sequencing of cloned *dnmt1 *** **fragments.** Click here for file

Additional file 12 **Results from sequencing of cloned *pou5f1 *** **fragments.** Click here for file

Additional file 13 All last exons with evidence for an extended 3’UTR post-ZGA relative to the pre-ZGA. Click here for file

Additional file 14 Different scripts used in the analyses. Click here for file

Additional file 15 A list with description of the primers. Click here for file
