# Supplementary material for: Differential transcript isoform usage pre- and post-zygotic genome activation in zebrafish
Source: BMC Genomics. 2013 May 15;14:331. doi: 10.1186/1471-2164-14-331 (PMC3747860; doi:10.1186/1471-2164-14-331)
Supplement: Additional file 1 — Supplementary figures 1–9. [file 1471-2164-14-331-S1.pdf]

Legends for figures in Additional file 1

Figure S1

Mapping of reads. Two different short-read aligner programs were used to map RNA-seq reads onto the zebrafish genome at pre- and post-MBT stages of development (representing pre- and post-ZGA transcriptomes). These two aligners map more reads when combined than when used individually.

Figure S2

IGV screenshot. TopHat and Bioscope complement each other; some reads were only mapped by TopHat, others only by Bioscope. Using both results in better coverage and improvement of the accuracy of the assembled isoforms. The arrows indicate Tophat (grey arrow) and Bioscope (white arrow) specific reads. In 'both' reads mapped by the two aligners are merged. The visualized area is on chromosome 1:396600-402000.

Figure S3

dazl isoform expression. *dazl* has two isoforms expressed maternally which exhibit differential degradation kinetics; isoform 2 is degraded more rapidly than isoform 1 and becomes the minor isoform after the 256-cell stage.

Figure S4

H3K4me3 marking of TSSs post-ZGA. **(a)** H3K4me3 post-translational modification marks (black bar) multiple alternative *dazl* TSSs (black arrows) at the post-ZGA stage. **(b)** Different

Sall4 TSSs are used pre-ZGA and post-ZGA. The maternal RNAs were transcribed from a TSS (black arrow) different from the zygotic transcripts (grey arrow). The H3K4me3 marks both TSSs (black bar). **(c)**_*Actl6a* is transcribed from different promoters. Maternal isoform (black arrow) and post-ZGA (grey arrow). Only the post-ZGA TSS is marked by K3K4me3 (black bar).

Figure S5

qPCR validation of *f11r*. qPCR results for *f11r* confirms the onset of expression of the isoform with exon 8 skipped at 5.5 hpf (red asterix).

Figure S6

Novel isoforms of *dnmt1*. Numerous novel isoforms for *dnmt1* were detected; several were produced due to skipping of multiple exons.

Figure S7

Novel isoforms of *pou5f1*. We detected several new AS events which led to the assembly of four isoforms (grey arrow) different from the annotated version. We also observed a longer 3’ UTR than in the annotated version (black arrow), in particular at the post-ZGA stage (white arrow).

Figure S8

Metagene of skipped exons calculated from RNA-seq reads. Red line = skipped exons. Blue line = random set of exons.

Figure S9

qPCR validation of *magi1*expression.

magi1 expresses a post-ZGA specific isoform (red asterix).

S1

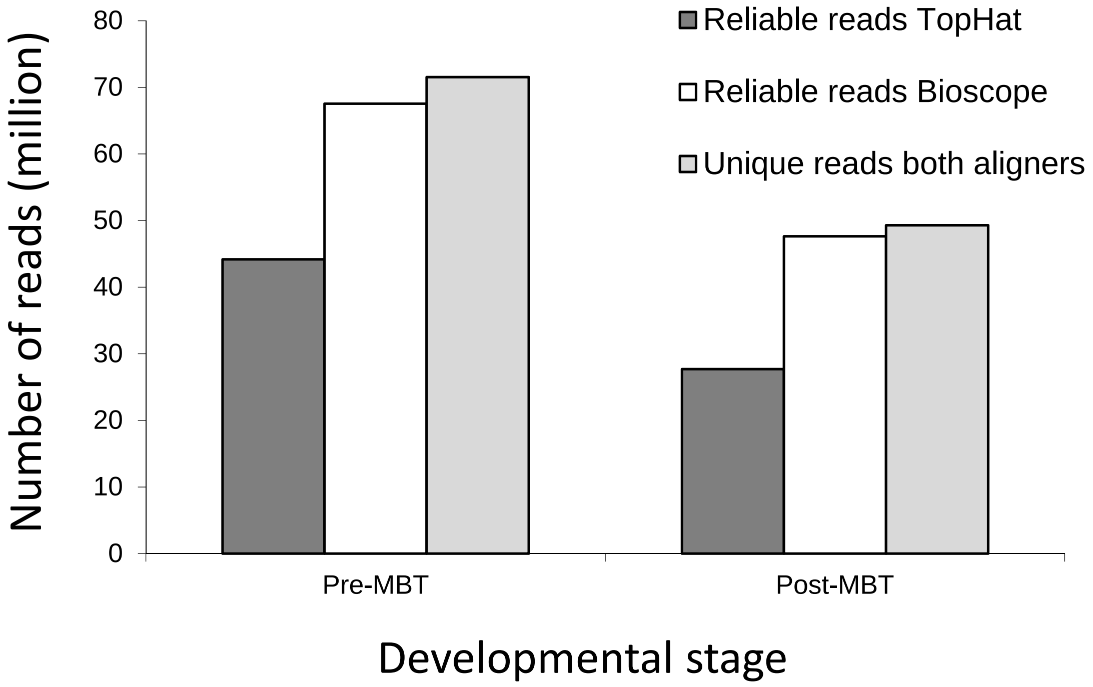
Number of reads (million)
0
10
20
30
40
50
60
70
80
Reliable reads TopHat
Reliable reads Bioscope
Unique reads both aligners
Pre-MBT
Post-MBT
Developmental stage

S2

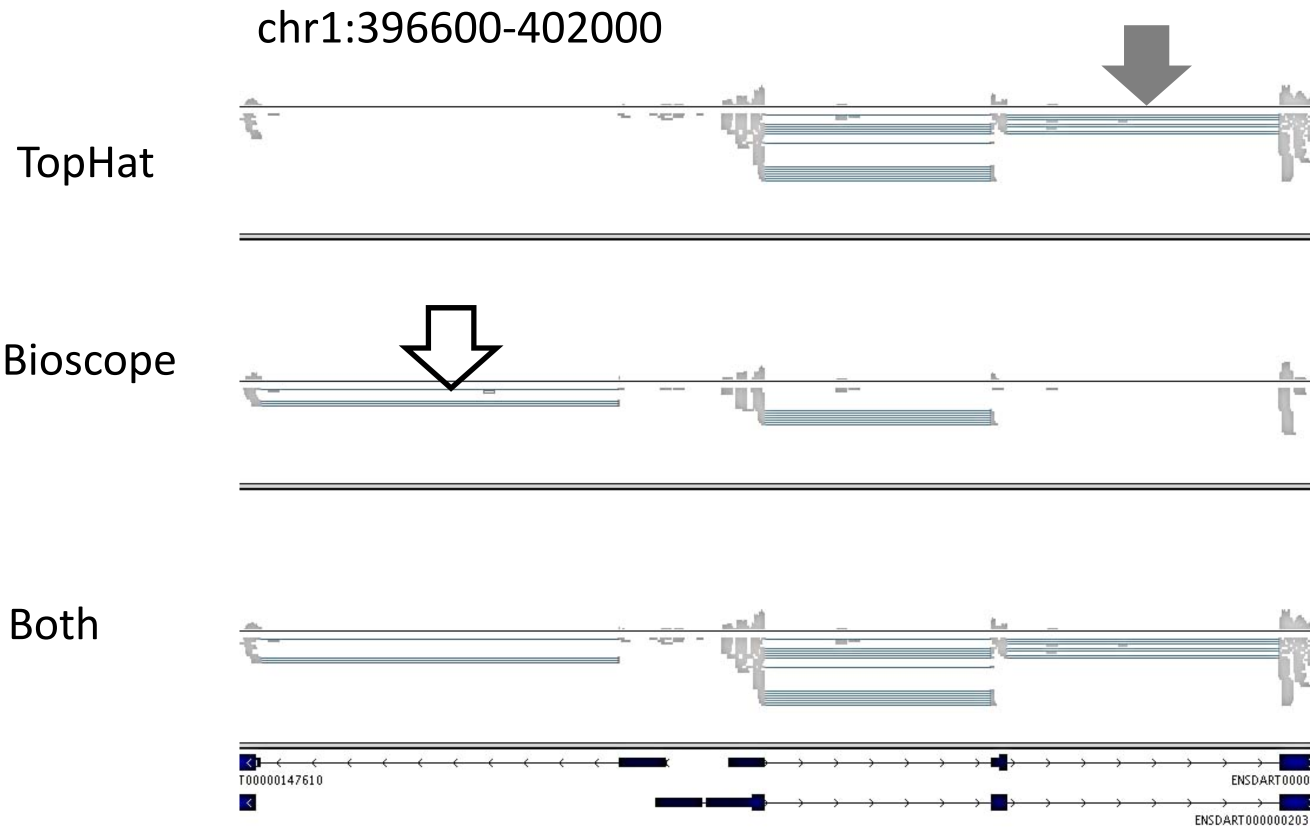
chr1:396600-402000
TopHat
Bioscope
Both
T00000147610
ENSDART0000
ENSDART000000203

S3

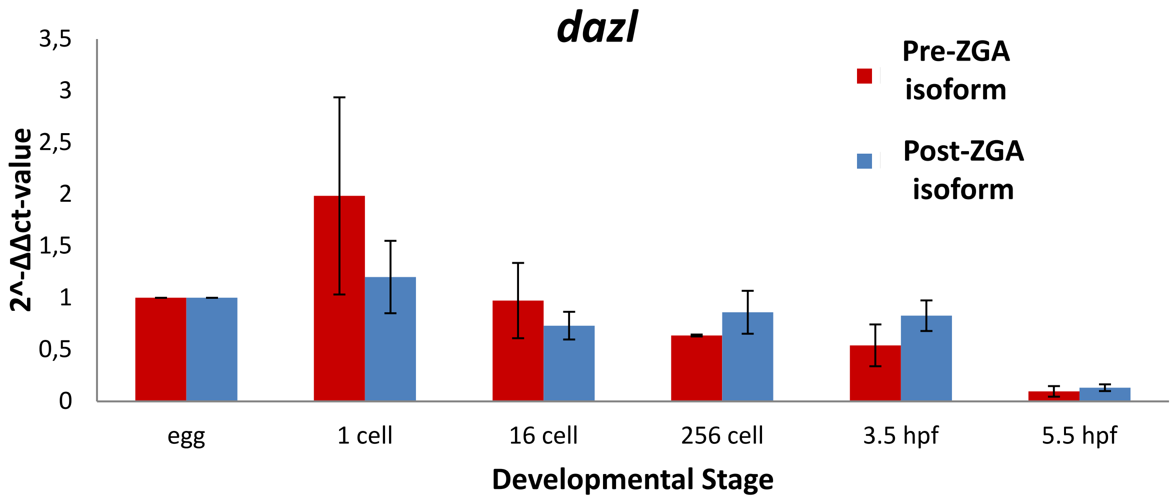
dazl
3,5
3
2,5
2
1,5
1
0,5
0
2^-ΔΔct-value
Pre-ZGA isoform
Post-ZGA isoform
egg
1 cell
16 cell
256 cell
3.5 hpf
5.5 hpf
Developmental Stage

S4

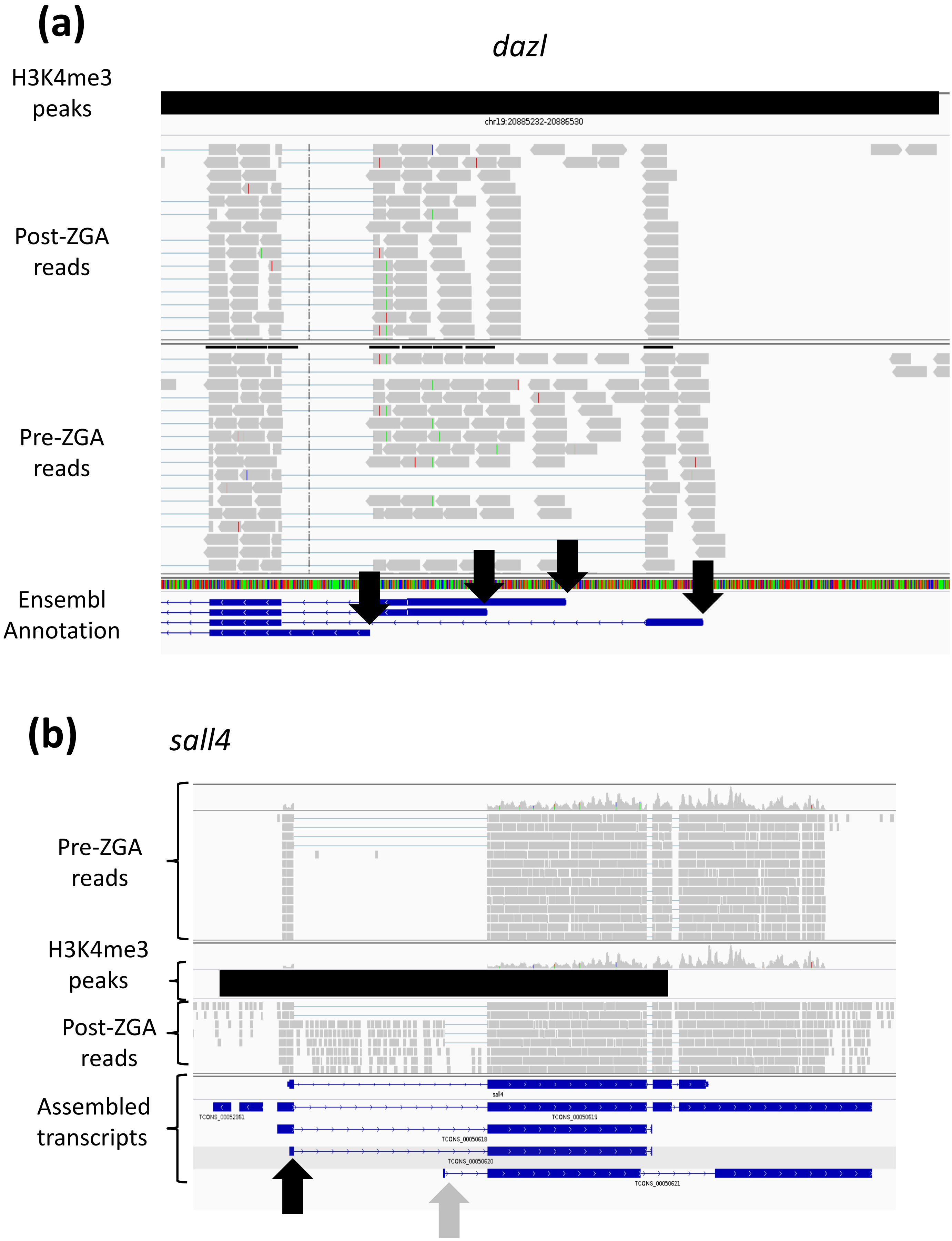
(a)
dazl
H3K4me3 peaks
chr19:20885232-20886530
Post-ZGA reads
Pre-ZGA reads
Ensembl Annotation
(b)
sall4
Pre-ZGA reads
H3K4me3 peaks
Post-ZGA reads
Assembled transcripts
sall4
TCONS_00052361
TCONS_00050619
TCONS_00050618
TCONS_00050620
TCONS_00050621

S4

(c)

actl6a

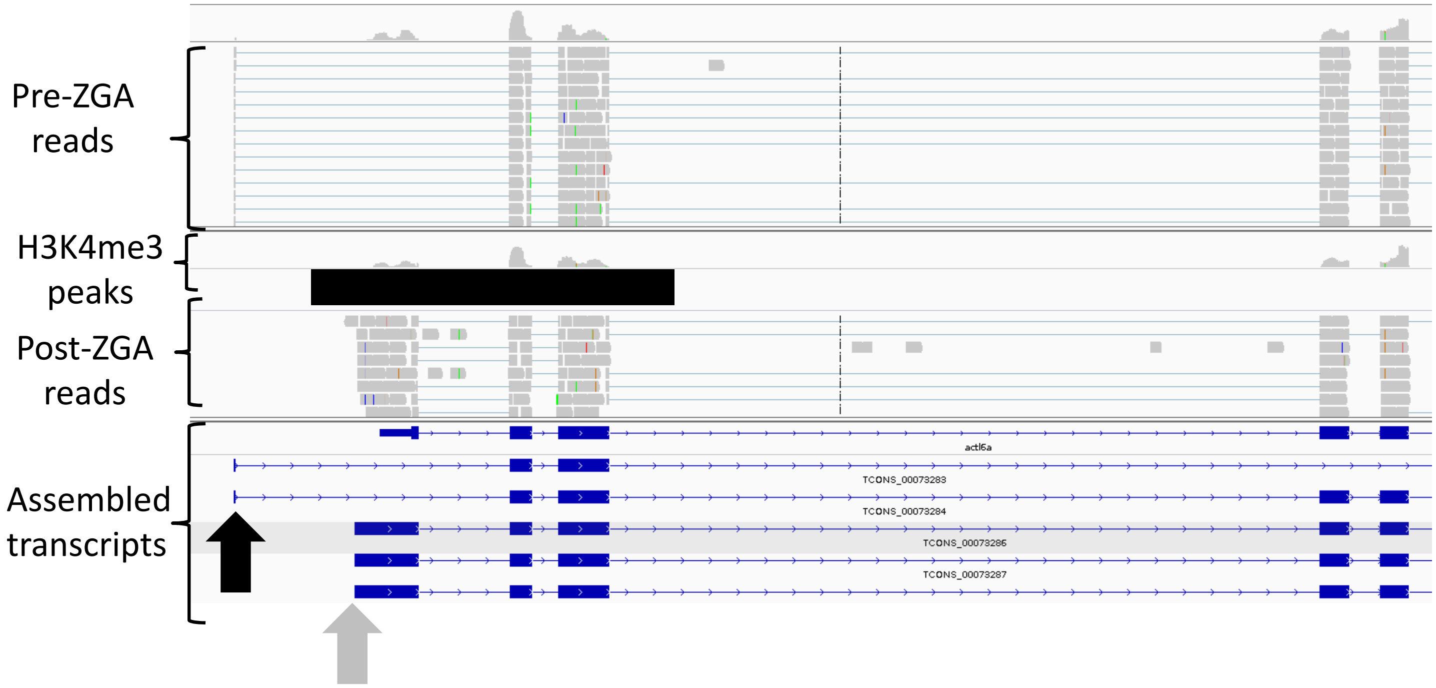

Pre-ZGA reads
H3K4me3 peaks
Post-ZGA reads
Assembled transcripts
actl6a
TCONS_00073283
TCONS_00073284
TCONS_00073286
TCONS_00073287

S5

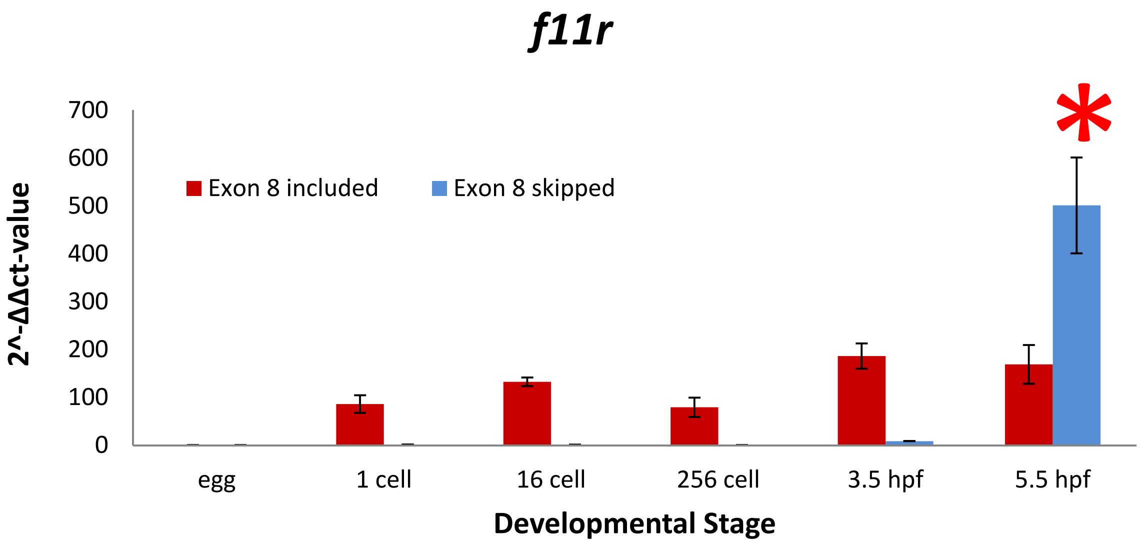
f11r
Exon 8 included
Exon 8 skipped
700
600
500
400
300
200
100
0
2^-ΔΔct-value
egg
1 cell
16 cell
256 cell
3.5 hpf
5.5 hpf
Developmental Stage
*

S6

dnmt1

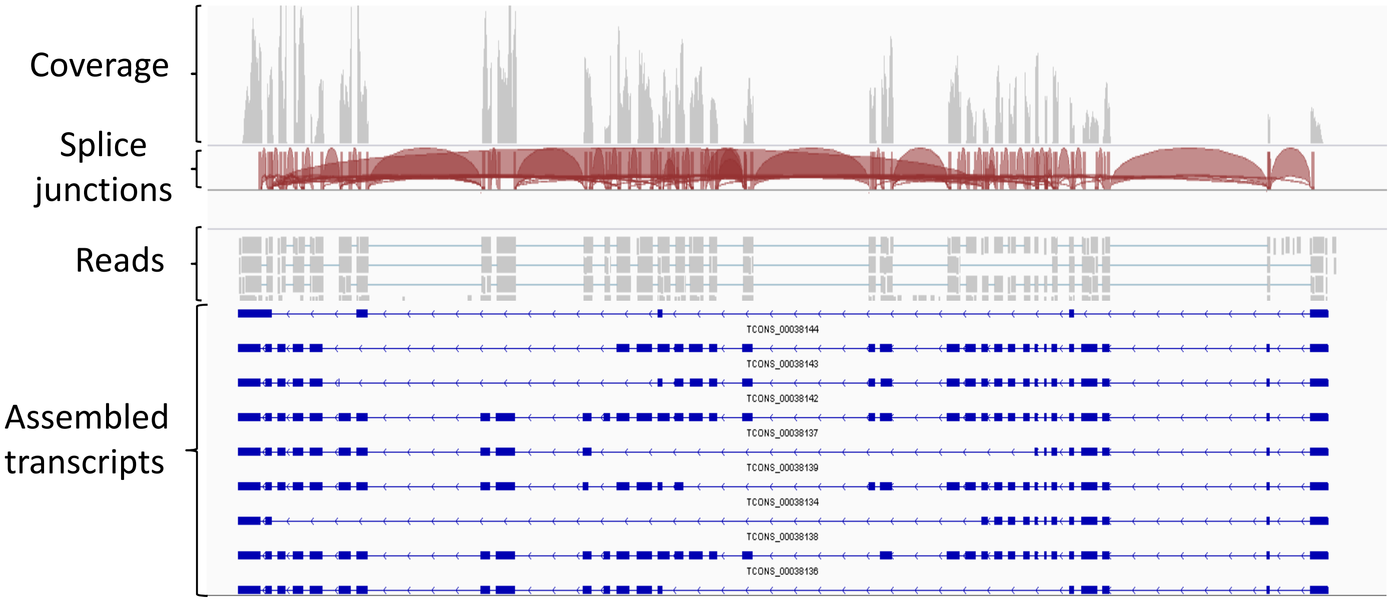

S7

pou5f1

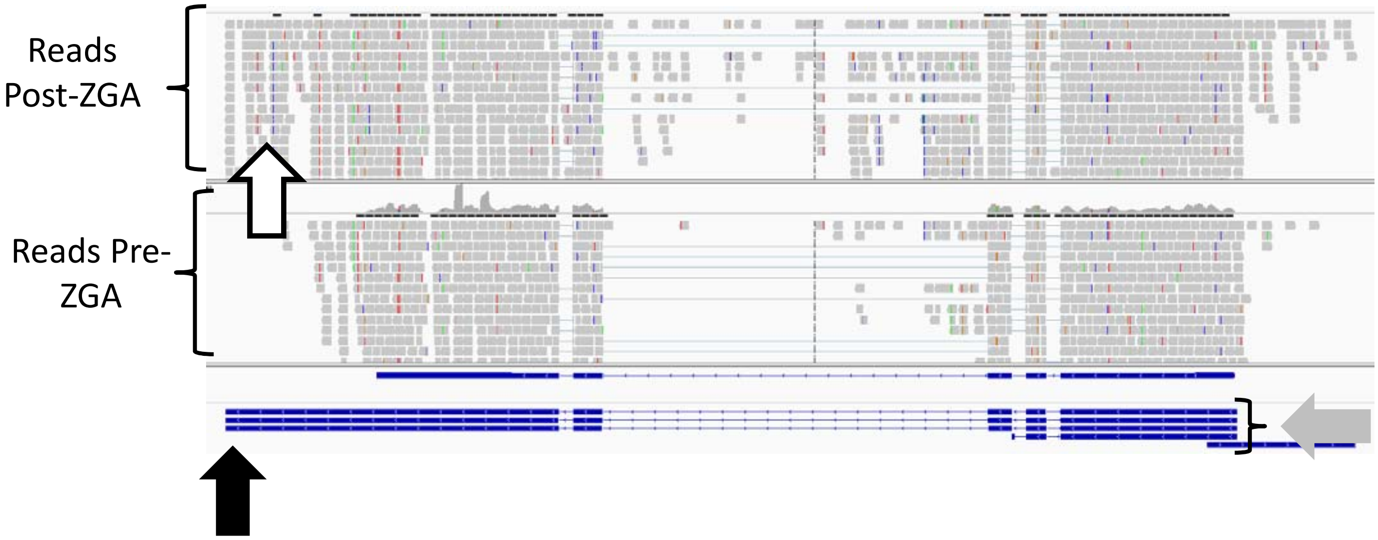

S8

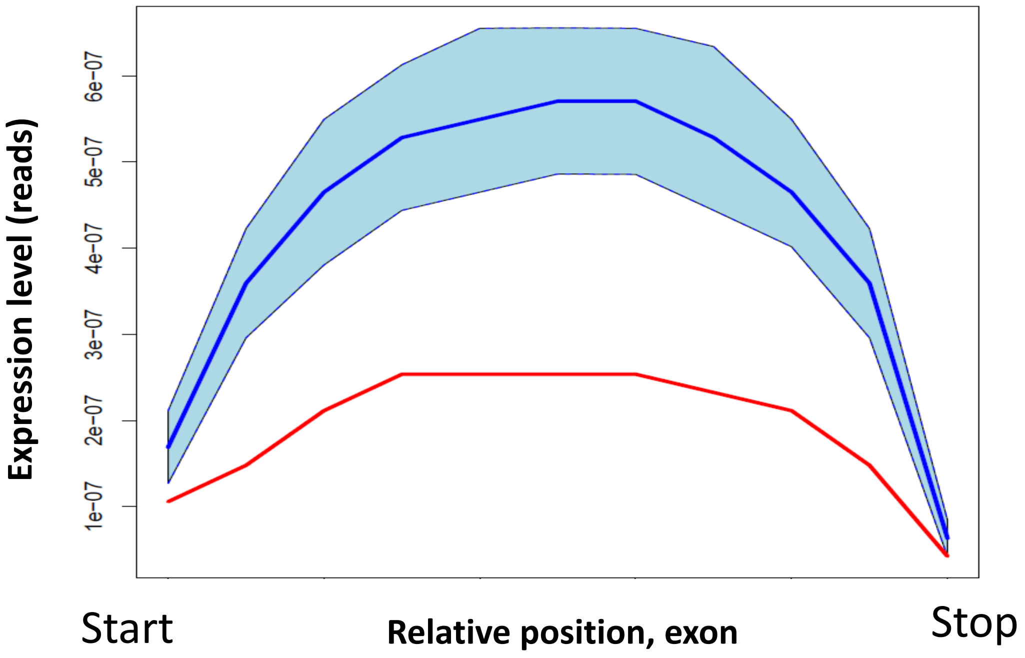
Expression level (reads)
1e-07
2e-07
3e-07
4e-07
5e-07
6e-07
Start
Relative position, exon
Stop

S9

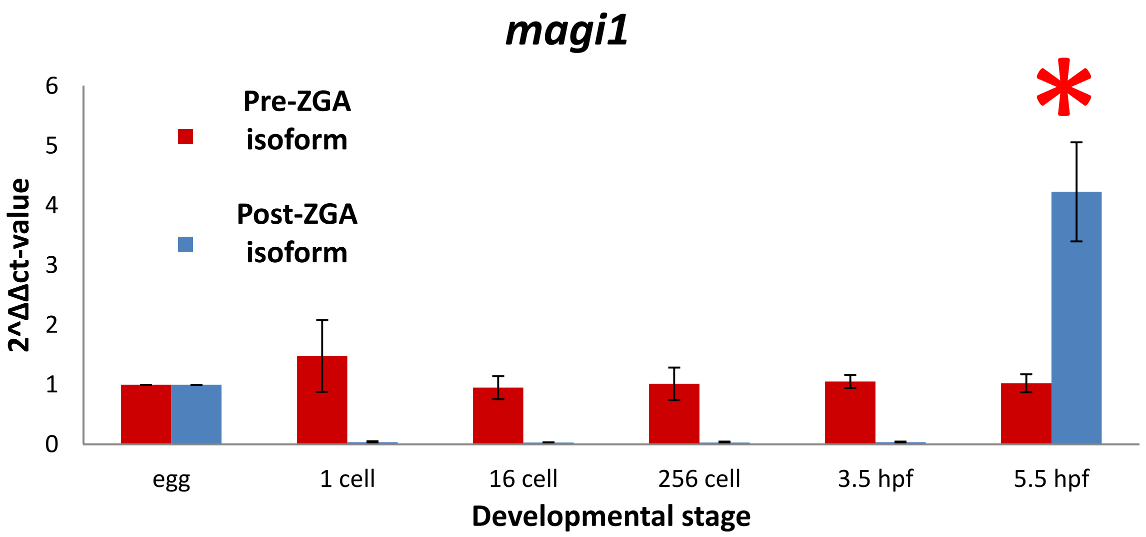
magi1
Pre-ZGA isoform
Post-ZGA isoform
*
2^ΔΔct-value
0
1
2
3
4
5
6
egg
1 cell
16 cell
256 cell
3.5 hpf
5.5 hpf
Developmental stage
