# Supplementary material for: Differential transcript isoform usage pre- and post-zygotic genome activation in zebrafish
Source: BMC Genomics. 2013 May 15;14:331. doi: 10.1186/1471-2164-14-331 (PMC3747860; doi:10.1186/1471-2164-14-331)
Supplement: Additional file 11 — Results from sequencing of cloned dnmt1 fragments. [file 1471-2164-14-331-S11.docx]

TCGGATCACTAGTAACGGCCGCCAGTGTGCTGGAATTCGCCCTTGGCTTCACTACAGGTT

GGTAATGCTGTTCCTCCACCCCTCTTTAAAGCCATTGGCCTGGAGGTCAAGAAGTGTGTG

CTGGAGAAAATGAGGGAGAATGCTACAGAGCCTGTGAAGCAGGAGAAAATGGAGCTCTCT

GACTAAAGCCATCTAAGGGCGAATTCTGCAGATATCCATCACACTGGCGGCCGCTCGAGC

ATGCATCTAGAGGGCCCAATTCGCCCTATAGTGAGTCGTATTACAATTCACTGGCCGTCG

TTTTACAACGTCGTGACTGGGAAAACCCTGGCGTTACCCAACTTAATCGCCTTGCAGCAC

ATCCCCCTTTCGCCAGCTGGCGTAATAGCGAAGAGGCCCGCACCGATCGCCCTTCCCAAC

AGTTGCGCAGCCTGAATGGCGAATGGACGCGCCCTGTAGCGGCGCATTAAGCGCGGCGGG

TGTGGTGGTTACGCGCAGCGTGACCGCTACACTTGCCAGCGCCCTAGCGCCCGCTCCTTT

CGCTTTCTTCCCTTCCTTTCTCGCCACGTTCGCCGGCTTTCCCCGTCAAGCTCTAAATCG

GGGGCTCCCTTTAGGGTTCCGATTTAGTGCTTTACGGCACCTCGACCCCAAAAAACTTGA

TTAGGGTGATGGTTCACGTAGTGGGCCATCGCCCTGATAGACGGTTTTTCGCCCTTTGAC

GTTGGAGTCCACGTTCTTTAATAGTGGACTCTTGTTCCAAACTGGAACAACACTCAACCC

TATCTCGGTCTATTCTTTTGATTTATAAGGGATTTTGCCGATTTCGGCCTATTGGTTAAA

AAATGAGCTGATTTAACAAAAATTTAACGCGAATTTTAACAAAATTCAGGGCGCAAGGGC

TGCTAAAGGAAGCGGAACACGTAGAAAGCCAGTCCGCAGAAACGGTGCTGACCCCGGATG

ATGTCAGCTACTGGGCTATCTGGACAAGGGAAAACGCAAGCGCAAAGAGAAAGCAGTAGC

T

CGGATCACTAGTAACGGCCGCCAGTGTGCTGGAATTCGCCCTTGGCTTCACTACAGGTTG

GTAATGCTGTTCCTCCACCCCTCCCTAAAGCCATTGGCCTGGAGGTCAAGAAGTGTGTGC

TGGAGAAAATGAGGGAGAATGCTACAGAGCCTGTGAAGCAGGAGAAAATGGAGCTCTCTG

ACTAAAGCCATCATAAGGGCGAATTCTGCAGATATCCATCACACTGGCGGCCGCTCGAGC

ATGCATCTAGAGGGCCCAATTCGCCCTATAGTGAGTCGTATTACAATTCACTGGCCGTCG

TTTTACAACGTCGTGACTGGGAAAACCCTGGCGTTACCCAACTTAATCGCCTTGCAGCAC

ATCCCCCTTTCGCCAGCTGGCGTAATAGCGAAGAGGCCCGCACCGATCGCCCTTCCCAAC

AGTTGCGCAGCCTGAATGGCGAATGGACGCGCCCTGTAGCGGCGCATTAAGCGCGGCGGG

TGTGGTGGTTACGCGCAGCGTGACCGCTACACTTGCCAGCGCCCTAGCGCCCGCTCCTTT

CGCTTTCTTCCCTTCCTTTCTCGCCACGTTCGCCGGCTTTCCCCGTCAAGCTCTAAATCG

GGGGCTCCCTTTAGGGTTCCGATTTAGTGCTTTACGGCACCTCGACCCCAAAAAACTTGA

TTAGGGTGATGGTTCACGTAGTGGGCCATCGCCCTGATAGACGGTTTTTCGCCCTTTGAC

GTTGGAGTCCACGTTCTTTAATAGTGGACTCTTGTTCCAACTGGAACAACACTCAACCCT

ATCTCGGTCTATTCTTTTGATTTATAAGGGATTTTGCCGATTTCGGCCTATTGGTTAAAA

AATGAGCTGATTTAACAAAAATTTAACGCGAATTTTAACAAAATTCACGGCGCAAGGGCT

GCTAAAGGAAGCGGAACACGTAGAAAGCCAGTCCGCAGAAACGGTGCTGACCCCGGATGA

ATGTCAGCTACTGGGCTATCTGGACAG

This one is the right one

TACGAGCTCGGATCCACTAGTAACGGCCGCCAGTGTGCTGGAATTCGCCCTTGGCTTCAC

TACAGGTTGGTAATGCTGTTCCTCCACCCCTCTCCAAAGCCATTGGCCTGGAGGTCAAGA

AGTGTGTGCTGGAGAAAATGAGGGAGAATGCTACAGAGCCTGTGAAGCAGGAGAAAATGG

AGCTCTCTGACTAAAGCCATCATAAGGGCGAATTCTGCAGATATCCATCACACTGGCGGC

CGCTCGAGCATGCATCTAGAGGGCCCAATTCGCCCTATAGTGAGTCGTATTACAATTCAC

TGGCCGTCGTTTTACAACGTCGTGACTGGGAAAACCCTGGCGTTACCCAACTTAATCGCC

TTGCAGCACATCCCCCTTTCGCCAGCTGGCGTAATAGCGAAGAGGCCCGCACCGATCGCC

CTTCCCAACAGTTGCGCAGCCTGAATGGCGAATGGACGCGCCCTGTAGCGGCGCATTAAG

CGCGGCGGGTGTGGTGGTTACGCGCAGCGTGACCGCTACACTTGCCAGCGCCCTAGCGCC

CGCTCCTTTCGCTTTCTTCCCTTCCTTTCTCGCCACGTTCGCCGGCTTTCCCCGTCAAGC

TCTAAATCGGGGGCTCCCTTTAGGGTTCCGATTTAGTGCTTTACGGCACCTCGACCCCAA

AAAACTTGATTAGGGTGATGGTTCACGTAGTGGGCCATCGCCCTGATAGACGGTTTTTCG

CCCTTTGACGTTGGAGTCCACGTTCTTTAATAGTGGACTCTTGTTCCAAACTGGAACAAC

ACTCAACCCTATCTCGGTCTATTCTTTTGATTTATAAGGGATTTTGCCGATTTCGGCCTA

TTGGTTAAAAAATGAGCTGATTTAACAAAAATTTAACGCGAATTTTAACAAAATTCAGGC

GCAAGGGCTGCTAAAGGAAGCGGAACACGTAGAAAGCCAGTCCGCAGAAACGGTGCTGAC

CCCGGATGAATGTCAGCTACTGGGCTATCTGGACAAGGGAAAACGCAAGCGCAAAGAGAA

AGCAGGTAGCTTGCAGTGGGCTTACATGG

TACGAGCTCGGATCCACTAGTAACGGCCGCCAGTGTGCTGGAATTCGCCCTTATGATGGC

TTTAGTCAGAGAGCTCCATTTTCTCCTGCTTCACAGGCTCTGTAGCATTCTCCCTCATTT

TCTCCAGCACACACTTCTTGACCTCCAGGCCAATGGCTTTGGAGAGGGGTGGAGGAACAG

CATTACCAACCTGTAGTGAAGCCAAGGGCGAATTCTGCAGATATCCATCACACTGGCGGC

CGCTCGAGCATGCATCTAGAGGGCCCAATTCGCCCTATAGTGAGTCGTATTACAATTCAC

TGGCCGTCGTTTTACAACGTCGTGACTGGGAAAACCCTGGCGTTACCCAACTTAATCGCC

TTGCAGCACATCCCCCTTTCGCCAGCTGGCGTAATAGCGAAGAGGCCCGCACCGATCGCC

CTTCCCAACAGTTGCGCAGCCTGAATGGCGAATGGACGCGCCCTGTAGCGGCGCATTAAG

CGCGGCGGGTGTGGTGGTTACGCGCAGCGTGACCGCTACACTTGCCAGCGCCCTAGCGCC

CGCTCCTTTCGCTTTCTTCCCTTCCTTTCTCGCCACGTTCGCCGGCTTTCCCCGTCAAGC

TCTAAATCGGGGGCTCCCTTTAGGGTTCCGATTTAGTGCTTTACGGCACCTCGACCCCAA

AAAACTTGATTAGGGTGATGGTTCACGTAGTGGGCCATCGCCCTGATAGACGGTTTTTCG

CCCTTTGACGTTGGAGTCCACGTTCTTTAATAGTGGACTCTTGTTCCAAACTGGAACAAC

ACTCAACCCTATCTCGGTCTATTCTTTTGATTTATAAGGGATTTTGCCGATTTCGGCCTA

TTGGTTAAAAAATGAGCTGATTTAACAAAAATTTAACGCGAATTTTAACAAAATTCAGGC

GCAAGGGCTGCTAAAGGAAGCGGAACACGTAGAAAGCCAGTCCGCAGAAACGGTGCTGAC

CCCGGATGATGTCAGCTACTGGGCTATCTGGACAGG

TACGAGCTCGGATCCACTAGTAACGGCCGCCAGTGTGCTGGAATTCGCCCTTGGCTTCAC

TACAGGTTGGTAATGCTGTTCCTCCACCCCTCTCCAAAGCCATTGGCCTGGAGGTCAAGA

AGTGTGTGCTGGAGAAAATGAGGGAGAATGCCACAGAGCCTGTGAAGCAGGAGAAAATGG

AGCTCTCTGACTAAAGCCATCATAAGGGCGAATTCTGCAGATATCCATCACACTGGCGGC

CGCTCGAGCATGCATCTAGAGGGCCCAATTCGCCCTATAGTGAGTCGTATTACAATTCAC

TGGCCGTCGTTTTACAACGTCGTGACTGGGAAAACCCTGGCGTTACCCAACTTAATCGCC

TTGCAGCACATCCCCCTTTCGCCAGCTGGCGTAATAGCGAAGAGGCCCGCACCGATCGCC

CTTCCCAACAGTTGCGCAGCCTGAATGGCGAATGGACGCGCCCTGTAGCGGCGCATTAAG

CGCGGCGGGTGTGGTGGTTACGCGCAGCGTGACCGCTACACTTGCCAGCGCCCTAGCGCC

CGCTCCTTTCGCTTTCTTCCCTTCCTTTCTCGCCACGTTCGCCGGCTTTCCCCGTCAAGC

TCTAAATCGGGGGCTCCCTTTAGGGTTCCGATTTAGTGCTTTACGGCACCTCGACCCCAA

AAAACTTGATTAGGGTGATGGTTCACGTAGTGGGCCATCGCCCTGATAGACGGTTTTTCG

CCCTTTGACGTTGGAGTCCACGTTCTTTAATAGTGGACTCTTGTTCCAAACTGGAACAAC

ACTCAACCCTATCTCGGTCTATTCTTTTGATTTATAAGGGATTTTGCCGATTTCGGCCTA

TTGGTTAAAAAATGAGCTGATTTAACAAAAATTTAACGCGAATTTTAACAAAATTCAGGG

CGCAAGGGCTGCTAAAGGAAGCGGAACACGTAGAAAGCCAGTCCGCAGAAACGGTGCTGA

CCCCGGATGATGTCAGCTACTGGGCTATCTGGACAAG

CCC different nc (compared to sequence 1)

CAT inserted nucleotide (compared to sequence 1)

AACT missing nucleotide (compared to sequence 1)

GTGAA strange

Sequence identical with predicted amplicon

GGCTTCACTACAGGTTGGTAATGCTGTTCCTCCACCCCTCTCCAAAGCCATTGGCCTGGAGGTCAAGAAGTGTGTGCTGGAGAAAATGAGGGAGAATGCTACAGAGCCTGTGAAGCAGGAGAAAATGGAGCTCTCTGACTAAAGCCATCAT

151 bp
