# Supplementary material for: Differential transcript isoform usage pre- and post-zygotic genome activation in zebrafish
Source: BMC Genomics. 2013 May 15;14:331. doi: 10.1186/1471-2164-14-331 (PMC3747860; doi:10.1186/1471-2164-14-331)
Supplement: Additional file 12 — Results from sequencing of cloned pou5f1 fragments. [file 1471-2164-14-331-S12.docx]

**Pouf1 after sequencing**

Output:

Pou5f1 was amplified using primers overlapping the area of inserts and deletions. 12 clones send to sequencing. From these:

- 6 are identical with **iso3&5**
- 1 is identical with **iso2**
- 5 are supposing **iso1**. However, in original sequence send by Håvard it should be insert of GTAG. Instead, sequencing confirmed change in the sequence ACAA (in iso 3&5) to **GCAG** in the predicted position.
- 1 is identical with **iso4**

**R37 (iso 3&5)**

ACTAGTAACGGCCGCCAGTGTGCTGGAATTCGCCCTTGCAGTGATTCTGAGGAAGAGGAG

ACTCTGACTACTGAAGATTTGGAGCAGTTTGCGAAAGAGCTTAAACACAAGCGCATCACT

CTGGGCTTCACGCAGGCAGATGTGGGACTCGCGCTTGGAAACTTGTATGGCAAAATGTTC

AGTCAGACGACAATCTGCCGCTTTGAGGCTCTCCAACTTAGTTTCAAGAACATGTGCAAA

CTGAAGCCGTTGTTGCAGAGGTGGTTGAACGAGGCCGAAAACTCCGAGAACCCTCAGGAT

ATGTACAAAATTGAACGGGTGTTTGTCGACACGCGAAAAAGAAAACGAAGGACCAGCTTG

GAAGGCACAGTCCGTTCTGCTCTAGAGTCGTACTTCGTGAAGTGCCCCAAACCCAACACT

CTGGAGATAACGCACATATCCGATGATCTAGGCCTGGAGAAAGGGCGAATTCTGCAGATA

TCCATCACACTGGCGGCCGCTCGAGCATGCATCTAGAGGGCCCAATTCGCCCTATAGTGA

GTCGTATTACAATTCACTGGCCGTCGTTTTACAACGTCGTGACTGGGAAAACCCTGGCGT

TACCCAACTTAATCGCCTTGCAGCACATCCCCCTTTCGCCAGCTGGCGTAATAGCGAAGA

GGCCCGCACCGATCGCCCTTCCCAACAGTTGCGCAGCCTGAATGGCGAATGGACGCGCCC

TGTAGCGGCGCATTAAGCGCGGCGGGTGTGGTGGTTACGCGCAGCGTGACCGCTACACTT

GCCAGCGCCCTAGCGCCCGCTCCTTTCGCTTTCTTCCCTTCCTTTCTCGCCACGTTCGCC

GGCTTTCCCCGTCAAGCTCTAAATCGGGGGCTCCCTTTAGGGTTCCGATTTAGTGCTTTA

CGGCACCTCGACCCCAAAAAACTTGATTAGGGTGATGGTTCACGTAGTGGGCCATCGCCC

TGATAGACGGTTTTCGCCCTTTGACGTTGGAGTCCACGTTCTTTAATAGTGGACTCTTGT

TCCAAACTGGAACAACACTCAACCCTATCT

**R38, reverse-compl- (iso 3&5)**

GCTCGGATCCACTAGTAACGGCCGCCAGTGTGCTGGAATTCGCCCTTTCTCCAGGCCTAG

ATCATCGGATATGTGCGTTATCTCCAGAGTGTTGGGTTTGGGGCACTTCACGAAGTACGA

CTCTAGAGCAGAACGGACTGTGCCTTCCAAGCTGGTCCTTCGTTTTCTTTTTCGCGTGTC

GACAAACACCCGTTCAATTTTGTACATATCCTGAGGGTTCTCGGAGTTTTCGGCCTCGTT

CAACCACCTCTGCAACAACGGCTTCAGTTTGCACATGTTCTTGAAACTAAGTTGGAGAGC

CTCAAAGCGGCAGATTGTCGTCTGACTGAACATTTTGCCATACAAGTTTCCAAGCGCGAG

TCCCACATCTGCCTGCGTGAAGCCCAGAGTGATGCGCTTGTGTTTAAGCTCTTTCGCAAA

CTGCTCCAAATCTTCAGTAGTCAGAGTCTCCTCTTCCTCAGAATCACTGCAAGGGCGAAT

TCTGCAGATATCCATCACACTGGCGGCCGCTCGAGCATGCATCTAGAGGGCCCAATTCGC

CCTATAGTGAGTCGTATTACAATTCACTGGCCGTCGTTTTACAACGTCGTGACTGGGAAA

ACCCTGGCGTTACCCAACTTAATCGCCTTGCAGCACATCCCCCTTTCGCCAGCTGGCGTA

ATAGCGAAGAGGCCCGCACCGATCGCCCTTCCCAACAGTTGCGCAGCCTGAATGGCGAAT

GGACGCGCCCTGTAGCGGCGCATTAAGCGCGGCGGGTGTGGTGGTTACGCGCAGCGTGAC

CGCTACACTTGCCAGCGCCCTAGCGCCCGCTCCTTTCGCTTTCTTCCCTTCCTTTCTCGC

CACGTTCGCCGGCTTTCCCCGTCAAGCTCTAAATCGGGGGCTCCCTTTAGGGTTCCGATT

TAGTGCTTTACGGCACCTCGACCCCAAAAACTTGATTAGGGTGATGGTTCACGTAGTGGG

CCATCGCCCTGATAGACGGTTTTCGCCCTTTGACGTTGGAGTCCACGTTCTTTAATAGTG

**R39, reverse-compl. (iso3&5)**

GAGCTCGGATCCACTAGTAACGGCCGCCAGTGTGCTGGAATTCGCCCTTTCTCCAGGCCT

AGATCATCGGATATGTGCGTTATCTCCAGAGTGTTGGGTTTGGGGCACTTCACGAAGTAC

GACTCTAGAGCAGAACGGACTGTGCCTTCCAAGCTGGTCCTTCGTTTTCTTTTTCGCGTG

TCGACAAACACCCGTTCAATTTTGTACATATCCTGAGGGTTCTCGGAGTTTTCGGCCTCG

TTCAACCACCTCTGCAACAACGGCTTCAGTTTGCACATGTTCTTGAAACTAAGTTGGAGA

GCCTCAAAGCGGCAGATTGTCGTCTGACTGAACATTTTGCCATACAAGTTTCCAAGCGCG

AGTCCCACATCTGCCTGCGTGAAGCCCAGAGTGATGCGCTTGTGTTTGAGCTCTTTCGCA

AACTGCTCCAAATCTTCAGTAGTCAGAGTCTCCTCTTCCTCAGAATCACTGCAAGGGCGA

ATTCTGCAGATATCCATCACACTGGCGGCCGCTCGAGCATGCATCTAGAGGGCCCAATTC

GCCCTATAGTGAGTCGTATTACAATTCACTGGCCGTCGTTTTACAACGTCGTGACTGGGA

AAACCCTGGCGTTACCCAACTTAATCGCCTTGCAGCACATCCCCCTTTCGCCAGCTGGCG

TAATAGCGAAGAGGCCCGCACCGATCGCCCTTCCCAACAGTTGCGCAGCCTGAATGGCGA

ATGGACGCGCCCTGTAGCGGCGCATTAAGCGCGGCGGGTGTGGTGGTTACGCGCAGCGTG

ACCGCTACACTTGCCAGCGCCCTAGCGCCCGCTCCTTTCGCTTTCTTCCCTTCCTTTCTC

GCCACGTTCGCCGGCTTTCCCCGTCAAGCTCTAAATCGGGGGCTCCCTTTAGGGTTCCGA

TTTAGTGCTTTACGGCACCTCGACCCCAAAAACTTGATTAGGGTGATGGTTCACGTAGTG

GGCCATCGCCCTGATAGACGGTTTTCGCCCTTTGACGTTGGAGTCCACGTTCTTTAATAG

TGGACTC

**R40, reverse-compl. (iso 1, GCAG is ACAA, C is G and C is T)**

CTCGGATCCACTAGTAACGGCCGCCAGTGTGCTGGAATTCGCCCTTTCTCCAGGCCTAGA

TCATCGGATATGTGCGTTATCTCCAGAGTGTTGGGTTTGGGGCACTTCACGAAGTACGAC

TCTAGAGCAGAACGCACTGTGCCTTCCAAGCTGGTCCTCCGTTTTCTTTTTCGCGTGTCG

ACAAACACCCGTTCAATTTTGTACATATCCTGAGGGTTCTCGGAGTTTTCGGCCTCGTTC

AACCACCTCTGCAGCAGCGGCTTCAGTTTGCACATGTTCTTGAAACTAAGTTGGAGAGCC

TCAAAGCGGCAGATTGTCGTCTGACTGAACATTTTGCCATACAAGTTTCCAAGCGCGAGT

CCCACATCTGCCTGCGTGAAGCCCAGAGTGATGCGCTTGTGTTTGAGCTCTTTCGCAAAC

TGCTCCAAATCTTCAGTAGTCAGAGTCTCCTCTTCCTCAGAATCACTGCAAGGGCGAATT

CTGCAGATATCCATCACACTGGCGGCCGCTCGAGCATGCATCTAGAGGGCCCAATTCGCC

CTATAGTGAGTCGTATTACAATTCACTGGCCGTCGTTTTACAACGTCGTGACTGGGAAAA

CCCTGGCGTTACCCAACTTAATCGCCTTGCAGCACATCCCCCTTTCGCCAGCTGGCGTAA

TAGCGAAGAGGCCCGCACCGATCGCCCTTCCCAACAGTTGCGCAGCCTGAATGGCGAATG

GACGCGCCCTGTAGCGGCGCATTAAGCGCGGCGGGTGTGGTGGTTACGCGCAGCGTGACC

GCTACACTTGCCAGCGCCCTAGCGCCCGCTCCTTTCGCTTTCTTCCCTTCCTTTCTCGCC

ACGTTCGCCGGCTTTCCCCGTCAAGCTCTAAATCGGGGGCTCCCTTTAGGGTTCCGATTT

AGTGCTTTACGGCACCTCGACCCCAAAAACTTGATTAGGGTGATGGTTCACGTAGTGGC

**R41, reverse-compl. (iso 3&5, T is C)**

CTCGGATCCACTAGTAACGGCCGCCAGTGTGCTGGAATTCGCCCTTTCTCCAGGCCTAGA

TCATCGGATATGTGCGTTATCTCCAGAGTGTTGGGTTTGGGGCACTTCACGAAGTACGAC

TCTAGAGCAGAACGGACTGTGCCTTCCAAGCTGGTCCTTCGTTTTCTTTTTCGCGTGTCG

ACAAACACCCGTTCAATTTTGTACATATCCTGAGGGTTCTCGGAGTTTTCGGCCTCGTTC

AACCACCTCTGCAACAACGGCTTCAGTTTGCACATGTTCTTGAAACTAAGTTGGAGAGCC

TCAAAGCGGCAGATTGTCGTCTGACTGAACATTTTGCCATACAAGTTTCCAAGCGCGAGT

CCCACATCTGCCTGCGTGAAGCTCAGAGTGATGCGCTTGTGTTTAAGCTCTTTCGCAAAC

TGCTCCAAATCTTCAGTAGTCAGAGTCTCCTCTTCCTCAGAATCACTGCAAGGGCGAATT

CTGCAGATATCCATCACACTGGCGGCCGCTCGAGCATGCATCTAGAGGGCCCAATTCGCC

CTATAGTGAGTCGTATTACAATTCACTGGCCGTCGTTTTACAACGTCGTGACTGGGAAAA

CCCTGGCGTTACCCAACTTAATCGCCTTGCAGCACATCCCCCTTTCGCCAGCTGGCGTAA

TAGCGAAGAGGCCCGCACCGATCGCCCTTCCCAACAGTTGCGCAGCCTGAATGGCGAATG

GACGCGCCCTGTAGCGGCGCATTAAGCGCGGCGGGTGTGGTGGTTACGCGCAGCGTGACC

GCTACACTTGCCAGCGCCCTAGCGCCCGCTCCTTTCGCTTTCTTCCCTTCCTTTCTCGCC

ACGTTCGCCGGCTTTCCCCGTCAAGCTCTAAATCGGGGGCTCCCTTTAGGGTTCCGATTT

AGTGCTTTACGGCACCTCGACCCCAAAAAACTTGATTAGGGTGATGGTTCACGTAGTGGG

CCATCGCCCTGATAGACGGTTTTCGCCCTTTGACGTTGGAGTCCACGTTCTTTAATAGTG

GAC

**R42 (iso 1, CTGC is TTGT original, CACC is TAAA)**

GAAGAGGAGACTCTGACTACTGAAGATTTGGAGCAGTTTGCGAAAGAGCTCAACCACAAG

CGCATCACTCTGGGCTTCACGCAGGCAGATGTGGGACTCGCGCTTGGAAACTTGTATGGC

AAAATGTTCAGTCAGACGACAATCTGCCGCTTTGAGGCTCTCCAACTTAGTTTCAAGAAC

ATGTGCAAACTGAAGCCGCTGCTGCAGAGGTGGTTGAACGAGGCCGAAAACTCCGAGAAC

CCTCAGGATATGTACAAAATTGAACGGGTGTTTGTCGACACGCGAAAAAGAAAACGAAGG

ACCAGCTTGGAAGGCACAGTCCGTTCTGCTCTAGAGTCGTACTTCGTGAAGTGCCCCAAA

CCCAACACTCTGGAGATAACGCACATATCCGATGATCTAGGCCTGGAGAAAGGGCGAATT

CTGCAGATATCCATCACACTGGCGGCCGCTCGAGCATGCATCTAGAGGGCCCAATTCGCC

CTATAGTGAGTCGTATTACAATTCACTGGCCGTCGTTTTACAACGTCGTGACTGGGAAAA

CCCTGGCGTTACCCAACTTAATCGCCTTGCAGCACATCCCCCTTTCGCCAGCTGGCGTAA

TAGCGAAGAGGCCCGCACCGATCGCCCTTCCCAACAGTTGCGCAGCCTGAATGGCGAATG

GACGCGCCCTGTAGCGGCGCATTAAGCGCGGCGGGTGTGGTGGTTACGCGCAGCGTGACC

GCTACACTTGCCAGCGCCCTAGCGCCCGCTCCTTTCGCTTTCTTCCCTTCCTTTCTCGCC

ACGTTCGCCGGCTTTCCCCGTCAAGCTCTAAATCGGGGGCTCCCTTTAGGGTTCCGATTT

AGTGCTTTACGGCACCTCGACCCCAAAAAACTTGATTAGGGTGATGGTTCACGTAGTGGG

CCATC

**R43, reverse-compl. (iso 4, 19 nt insert; GCAG is ACAA)**

TCGGATCCACTAGTAACGGCCGCCAGTGTGCTGGAATTCGCCCTTTCTCCAGGCCTAGAT

CATCGGATATGTGCGTTATCTCCAGAGTGTTGGGTTTGGGGCACTTCACGAAGTACGACT

CTAGAGCAGAACGCACTGTGCCTTCCAAGCTGGTCCTCCGTTTTCTTTTTCGCGTGTCGA

CAAACACCCGTTCAATTTTGTACATATCCTGAGGGTTCTCGGAGTTTTCGGCCTCGTTCA

ACCACCTCTGCAGCAGCGGCTTCAGTTTGCACATGTTCTTGAAACTAAGTTGGAGAGCCT

CAAAGCGGCAGATTGTCGTCTGACTGAACATTTTGCCTTAAACAGAAAACACAACCATAC

AAGTTTCCAAGCGCGAGTCCCACATCTGCCTGCGTGAAGCCCAGAGTGATGCGCTTGTGT

TTGAGCTCTTTCGCAAACTGCTCCAAATCTTCAGTAGTCAGAGTCTCCTCTTCCTCAGAA

TCACTGCAAGGGCGAATTCTGCAGATATCCATCACACTGGCGGCCGCTCGAGCATGCATC

TAGAGGGCCCAATTCGCCCTATAGTGAGTCGTATTACAATTCACTGGCCGTCGTTTTACA

ACGTCGTGACTGGGAAAACCCTGGCGTTACCCAACTTAATCGCCTTGCAGCACATCCCCC

TTTCGCCAGCTGGCGTAATAGCGAAGAGGCCCGCACCGATCGCCCTTCCCAACAGTTGCG

CAGCCTGAATGGCGAATGGACGCGCCCTGTAGCGGCGCATTAAGCGCGGCGGGTGTGGTG

GTTACGCGCAGCGTGACCGCTACACTTGCCAGCGCCCTAGCGCCCGCTCCTTTCGCTTTC

TTCCCTTCCTTTCTCGCCACGTTCGCCGGCTTTCCCCGTCAAGCTCTAAATCGGGGGCTC

CCTTTAGGGTTCCGATTTAGTGCTTTACGGCACCTCGACCCCAAAAACTTGATTAGGGTG

ATGGTTCACGTAGTGGCC

R44

ATCCACTAGTAACGGCCGCCAGTGTGCTGGAATTCGCCCTTGCAGTGATTCTGAGGAAGA

GGAGACTCTGACTACTGAAGATTTGGAGCAGTTTGCGAAAGAGCTCAAACACAAGCGCAT

CACTCTGGGCTTCACGCAGGCAGATGTGGGACTCGCGCTTGGAAACTTGTATGGCAAAAT

GTTCAGTCAGACGACAATCTGCCGCTTTGAGGCTCTCCAACTTAGTTTCAAGAACATGTG

CAAACTGAAGCCGCTGCTGCAGAGGTGGTTGAACGAGGCCGAAAACTCCGAGAACCCTCA

GGATATGTACAAAATTGAACGGGTGTTTGTCGACACGCGAAAAAGAAAACGAAGGACCAG

CTTGGAAGGCACAGTCCGTTCTGCTCTAGAGTCGTACTTCGTGAAGTGCCCCAAACCCAA

CACTCTGGAGATAACGCACATATCCGATGATCTAGGCCTGGAGAAAGGGCGAATTCTGCA

GATATCCATCACACTGGCGGCCGCTCGAGCATGCATCTAGAGGGCCCAATTCGCCCTATA

GTGAGTCGTATTACAATTCACTGGCCGTCGTTTTACAACGTCGTGACTGGGAAAACCCTG

GCGTTACCCAACTTAATCGCCTTGCAGCACATCCCCCTTTCGCCAGCTGGCGTAATAGCG

AAGAGGCCCGCACCGATCGCCCTTCCCAACAGTTGCGCAGCCTGAATGGCGAATGGACGC

GCCCTGTAGCGGCGCATTAAGCGCGGCGGGTGTGGTGGTTACGCGCAGCGTGACCGCTAC

ACTTGCCAGCGCCCTAGCGCCCGCTCCTTTCGCTTTCTTCCCTTCCTTTCTCGCCACGTT

CGCCGGCTTTCCCCGTCAAGCTCTAAATCGGGGGCTCCCTTTAGGGTTCCGATTTAGTGC

TTTACGGCACCTCGACCCCAAAAACTTGATTAGGGTGATGGTTCACGTAGTGGGCCATCG

CCCTGATAGACGGTTTTTCGCCCTTTGACGTTGGAGTCACGTTCTTTAAT

R45

CGGATCCACTAGTAACGGCCGCCAGTGTGCTGGAATTCGCCCTTTCTCCAGGCCTAGATC

ATCGGATATGTGCGTTATCTCCAGAGTGTTGGGTTTGGGGCACTTCACGAAGTACGACTC

TAGAGCAGAACGGACTGTGCCTTCCAAGCTGGTCCTTCGTTTTCTTTTTCGCGTGTCGAC

AAACACCCGTTCAATTTTGTACATATCCTGAGGGTTCTCGGAGTTTTCGGCCTCGTTCAA

CCACCTCTGCAACAACGGCTTCAGTTTGCACATGTTCTTGAAACTAAGTTGGAGAGCCTC

AAAGCGGCAGATTGTCGTCTGACTGAACATTTTGCCGTACAAGTTTCCAAGCGCGAGTCC

CACATCTGCCTGCGTGAAGCCCAGAGTGATGCGCTTGTGTTTAAGCTCTTTCGCAAACTG

CTCCAAATCTTCAGTAGTCAGAGTCTCCTCTTCCTCAGAATCACTGCAAGGGCGAATTCT

GCAGATATCCATCACACTGGCGGCCGCTCGAGCATGCATCTAGAGGGCCCAATTCGCCCT

ATAGTGAGTCGTATTACAATTCACTGGCCGTCGTTTTACAACGTCGTGACTGGGAAAACC

CTGGCGTTACCCAACTTAATCGCCTTGCAGCACATCCCCCTTTCGCCAGCTGGCGTAATA

GCGAAGAGGCCCGCACCGATCGCCCTTCCCAACAGTTGCGCAGCCTGAATGGCGAATGGA

CGCGCCCTGTAGCGGCGCATTAAGCGCGGCGGGTGTGGTGGTTACGCGCAGCGTGACCGC

TACACTTGCCAGCGCCCTAGCGCCCGCTCCTTTCGCTTTCTTCCCTTCCTTTCTCGCCAC

GTTCGCCGGCTTTCCCCGTCAAGCTCTAAATCGGGGGGCTCCCTTTAGGGTTCCGATTTA

GTGCTTTACGGCACCTCGACCCCAAAAAACTTGATTAGGGT

R46

GGATCCACTAGTAACGGCCGCCAGTGTGCTGGAATTCGCCCTTTCTCCAGGCCTAGATCA

TCGGATATGTGCGTTATCTCCAGAGTGTTGGGTTTGGGGCACTTCACGAAGTACGACTCT

AGAGCAGAACGGACTGTGCCTTCCAAGCTGGTCCTTCGTTTTCTTTTTCGCGTGTCGACA

AACACCCGTTCAATTTTGTACATATCCTGAGGGTTCTCGGAGTTTTCGGCCTCGTTCAAC

CACCTCTGCAGCAGCGGCTTCAGTTTGCACATGTTCTTGAAACTAAGTTGGAGAGCCTCA

AAGCGGCAGATTGTCGTCTGACTGAACATTTTGCCATACAAGTTTCCAAGCGCGAGTCCC

ACATCTGCCTGCGTGAAGCCCAGAGTGATGCGCTTGTGTTTAAGCTCTTTCGCAAACTGC

TCCAAATCTTCAGTAGTCAGAGTCTCCTCTTCCTCAGAATCACTGCAAGGGCGAATTCTG

CAGATATCCATCACACTGGCGGCCGCTCGAGCATGCATCTAGAGGGCCCAATTCGCCCTA

TAGTGAGTCGTATTACAATTCACTGGCCGTCGTTTTACAACGTCGTGACTGGGAAAACCC

TGGCGTTACCCAACTTAATCGCCTTGCAGCACATCCCCCTTTCGCCAGCTGGCGTAATAG

CGAAGAGGCCCGCACCGATCGCCCTTCCCAACAGTTGCGCAGCCTGAATGGCGAATGGAC

GCGCCCTGTAGCGGCGCATTAAGCGCGGCGGGTGTGGTGGTTACGCGCAGCGTGACCGCT

ACACTTGCCAGCGCCCTAGCGCCCGCTCCTTTCGCTTTCTTCCCTTCCTTTCTCGCCACG

TTCGCCGGCTTTCCCCGTCAAGCTCTAAATCGGGGGCTCCCTTTAGGGTTCCGATTTAGT

GCTTTACGGCACCTCGACCCCAAAAAACTTGATTAGGGTGATGGTTCACGTAGTGGCC

R47

GAGCTCGGATCCACTAGTAACGGCCGCCAGTGTGCTGGAATTCGCCCTTGCAGTGATTCT

GAGGAAGAGGCTCTGACTACTGAAGATTTGGAGCAGTTTGCGAAAGAGCTTAAACACAAG

CGCATCACTCTGGGCTTCACGCAGGCAGATGTGGGACTCGCGCTTGGAAACTTGTATGGC

AAAATGTTCAGTCAGACGACAATCTGCCGCTTTGAGGCTCTCCAACTTAGTTTCAAGAAC

ATGTGCAAACTGAAGCCGTTGTTGCAGAGGTGGTTGAACGAGGCCGAAAACTCCGAGAAC

CCTCAGGATATGTACAAAATTGAACGGGTGTTTGTCGACACGCGAAAAAGAAAACGGAGG

ACCAGCTTGGAAGGCACAGTGCGTTCTGCTCTAGAGTCGTACTTCGTGAAGTGCCCCAAA

CCCAACACTCTGGAGATAACGCACATATCCGATGATCTAGGCCTGGAGAAAGGGCGAATT

CTGCAGATATCCATCACACTGGCGGCCGCTCGAGCATGCATCTAGAGGGCCCAATTCGCC

CTATAGTGAGTCGTATTACAATTCACTGGCCGTCGTTTTACAACGTCGTGACTGGGAAAA

CCCTGGCGTTACCCAACTTAATCGCCTTGCAGCACATCCCCCTTTCGCCAGCTGGCGTAA

TAGCGAAGAGGCCCGCACCGATCGCCCTTCCCAACAGTTGCGCAGCCTGAATGGCGAATG

GACGCGCCCTGTAGCGGCGCATTAAGCGCGGCGGGTGTGGTGGTTACGCGCAGCGTGACC

GCTACACTTGCCAGCGCCCTAGCGCCCGCTCCTTTCGCTTTCTTCCCTTCCTTTCTCGCC

ACGTTCGCCGGCTTTCCCCGTCAAGCTCTAAATCGGGGGCTCCCTTTAGGGTTCCGATTT

AGTGCTTTACGGCACCTCGACCCCAAAAAACTTGATTATGGTGAT

R48

TACGAGCTCGGATCCACTAGTAACGGCCGCCAGTGTGCTGGAATTCGCCCTTTCTCCAGG

CCTAGATCATCGGATATGTGCGTTATCTCCAGAGTGTTGGGTTTGGGGCACTTCACGAAG

TACGACTCTAGAGCAGAACGGACTGTGCCTTCCAAGCTGGTCCTTCGTTTTCTTTTTCGC

GTGTCGACAAACACCCGTTCAATTTTGTACATATCCTGAGGGTTCTCGGAGTTTTCGGCC

TCGTTCAACCACCTCTGCAACAACGGCTTCAGTTTGCACATGTTCTTGAAACTAAGTTGG

AGAGCCTCAAAGCGGCAGATTGTCGTCTGACTGAACATTTTGCCATACAAGTTTCCAAGC

GCGAGTCCCACATCTGCCTGCGTGAAGCCCAGAGTGATGCGCTTGTGTTTAAGCTCTTTC

GCAAACTGCTCCAAATCTTCAGTAGTCAGAGTCTCCTCTTCCTCAGAATCACTGCAAGGG

CGAATTCTGCAGATATCCATCACACTGGCGGCCGCTCGAGCATGCATCTAGAGGGCCCAA

TTCGCCCTATAGTGAGTCGTATTACAATTCACTGGCCGTCGTTTTACAACGTCGTGACTG

GGAAAACCCTGGCGTTACCCAACTTAATCGCCTTGCAGCACATCCCCCTTTCGCCAGCTG

GCGTAATAGCGAAGAGGCCCGCACCGATCGCCCTTCCCAACAGTTGCGCAGCCTGAATGG

CGAATGGACGCGCCCTGTAGCGGCGCATTAAGCGCGGCGGGTGTGGTGGTTACGCGCAGC

GTGACCGCTACACTTGCCAGCGCCCTAGCGCCCGCTCCTTTCGCTTTCTTCCCTTCCTTT

CTCGCCACGTTCGCCGGCTTTCCCCGTCAAGCTCTAAATCGGGGGCTCCCTTTAGGGTTC

CGATTTAGTGCTTTACGGCACCTCGACCCCAAAAACTTGATTAGGGTGATGGTTCACGTA

GTGGGCCATCGCCCTGATAGACGGTTTTCGCCCTTTGACGTTGGAGTC
